# Engineered isopeptide bond stabilized fibrin inspired nanoscale peptide based sealants for efficient blood clotting

**DOI:** 10.1038/s41598-017-06360-3

**Published:** 2017-07-26

**Authors:** Snehasish Ghosh, Sanchita Mukherjee, Chiranjit Dutta, Kasturee Chakraborty, Paramita Gayen, Somnath Jan, Dhananjay Bhattacharyya, Rituparna Sinha Roy

**Affiliations:** 1 Department of Chemical Sciences, Indian Institute of Science Education and Research Kolkata, Mohanpur, 741246 India; 2 Department of Biological Sciences, Indian Institute of Science Education and Research Kolkata, Mohanpur, 741246 India; 3 Computational Science Division, Saha Institute of Nuclear Physics, Kolkata, 1/AF Bidhannagar, Kolkata, 700064 India

## Abstract

Designing biologically inspired nanoscale molecular assembly with desired functionality is a challenging endeavour. Here we report the designing of fibrin-inspired nanostructured peptide based sealants which facilitate remarkably fast entrapping of blood corpuscles (~28 seconds) in contrast to fibrin (~56 seconds). Our engineered sealants are stabilized by lysine-aspartate ionic interactions and also by N^ε^(γ-glutamyl) lysine isopeptide bond mediated covalent interaction. Each sealant is formed by two peptides having complementary charges to promote lysine-aspartate ionic interactions and designed isopeptide bond mediated interactions. Computational analysis reveals the isopeptide bond mediated energetically favourable peptide assemblies in sealants 1–3. Our designed sealants 2 and 3 mimic fibrin-mediated clot formation mechanism in presence of transglutaminase enzyme and blood corpuscles. These fibrin-inspired peptides assemble to form sealants having superior hemostatic activities than fibrin. Designed sealants feature mechanical properties, biocompatibility, biodegradability and high adhesive strength. Such nature-inspired robust sealants might be potentially translated into clinics for facilitating efficient blood clotting to handle traumatic coagulopathy and impaired blood clotting.

## Introduction

Molecular assembly serves as emerging paradigm for engineering clinically important diverse nanostructured materials^1–3^. Such materials could be biologically inspired with desired functionality. However, *de novo* designing of nature inspired nanostructured material is indeed challenging, since it requires rational engineering of building blocks for self-assembled system and imparting desired functionality to it^1^.

In the living system, fibrinogen self-assembles into fibrin forming nanofibre-like morphology and clots whole blood as a part of blood clotting sequence^4^. Inspired by fibrin self-assembly mechanism and fibrin biology, we aim to design nature-inspired advanced functional biomaterials which could facilitate blood clotting more efficiently than fibrin. Ideally, such nature-inspired sealants should be biocompatible and biodegradable with minimal inflammatory response^5–13^. The natural process of blood clotting involves entrapment of blood corpuscles and platelets by fibrin^14, 15^. Fibrin is formed due to thrombin mediated polymerization and factor XIIIa induced intermolecular isopeptide bond mediated cross-linking of fibrinogen^4, 16–21^. In presence of transglutaminase enzyme (TG), ε-amino group of lysine residue of one protein and side-chain carboxyamide group of glutamine of another protein or same protein forms isopeptide bond via transient thioester intermediate^22–24^. Such intermolecular or intramolecular isopeptide bond mediated posttranslational modification provides thermal, mechanical and proteolytic stability to proteins^24^.

In this study, we have designed isopeptide bond stabilized fibrin-inspired peptide based sealants which can effectively induce blood-clotting and have performed biophysical studies to characterize the sealants and *ex-vivo* clotting studies to evaluate its efficacy. Sealant 1 is formed by the self-assembly of the functional segment of human fibrinogen γ-chain, stabilized by N^ε^(γ-glutamyl) lysine isopeptide bond. Sealants 2 and 3 are formed by the two peptides having complementary charges to promote lysine-aspartate ionic interactions^25^ and designed isopeptide bond mediated interactions^21–24^. ^D^Ala residue was introduced as spacer between the two adjacent ionic residues in peptide sequences forming sealant 3 to minimize adjacent Lys-Lys and Asp-Asp repulsion^26^. Insertion of ^D^Ala residues imparts proteolytic stability to the sealant 3^26^ and also allows us to probe how the change in orientation of isopeptide bond can affect the sealant stability in case of sealants 2 and 3. Our hypothesis was lysine-aspartate ionic interactions and appropriate orientation of isopeptide bond mediated interactions in designed peptides would result in superior hemostatic activity in designed sealants compared to the fibrin. Our studies demonstrate that engineered sealants 2 and 3 can successfully entrap blood corpuscles like fibrin and facilitate blood coagulation more efficiently than the isolated functional segment of fibrinogen and even fibrin.

Efficient blood clotting remains the most effective strategy for treating combat and accident casualties and also hemophilia patients^5, 27, 28^. Although a number of hemostatic agents are used to treat bleeding wounds depending on the location and the type of the injury, all of them have certain disadvantages^6^. Traumatic wounds cause coagulopathic conditions due to reduced blood flow and enhanced degradation of fibrin^14, 29^. Such impaired blood clotting cause huge number of deaths of civilian trauma patients^30^ and military mortality^14^. Generating rapid hemostasis from nature-inspired biodegradable hemostat material for such coagulopathic conditions will be advantageous. Such hemostat materials are required to facilitate blood clotting through a mechanism independent of body’s own blood coagulation pathway to handle traumatic coagulopathy^14^.

To address the challenges of coagulopathy, an effective approach to facilitate rapid blood-clotting will be the design of biodegradable peptide based hemostat. Such biomaterials designed and evaluated in our present study might be potentially translated into clinics especially to treat traumatic coagulopathy^31^ or impaired blood clotting conditions^28^ and are expected to meet the medical challenges^5–14^.

## Results and Discussion

### Nanostructured peptide design

**Figure 1:**
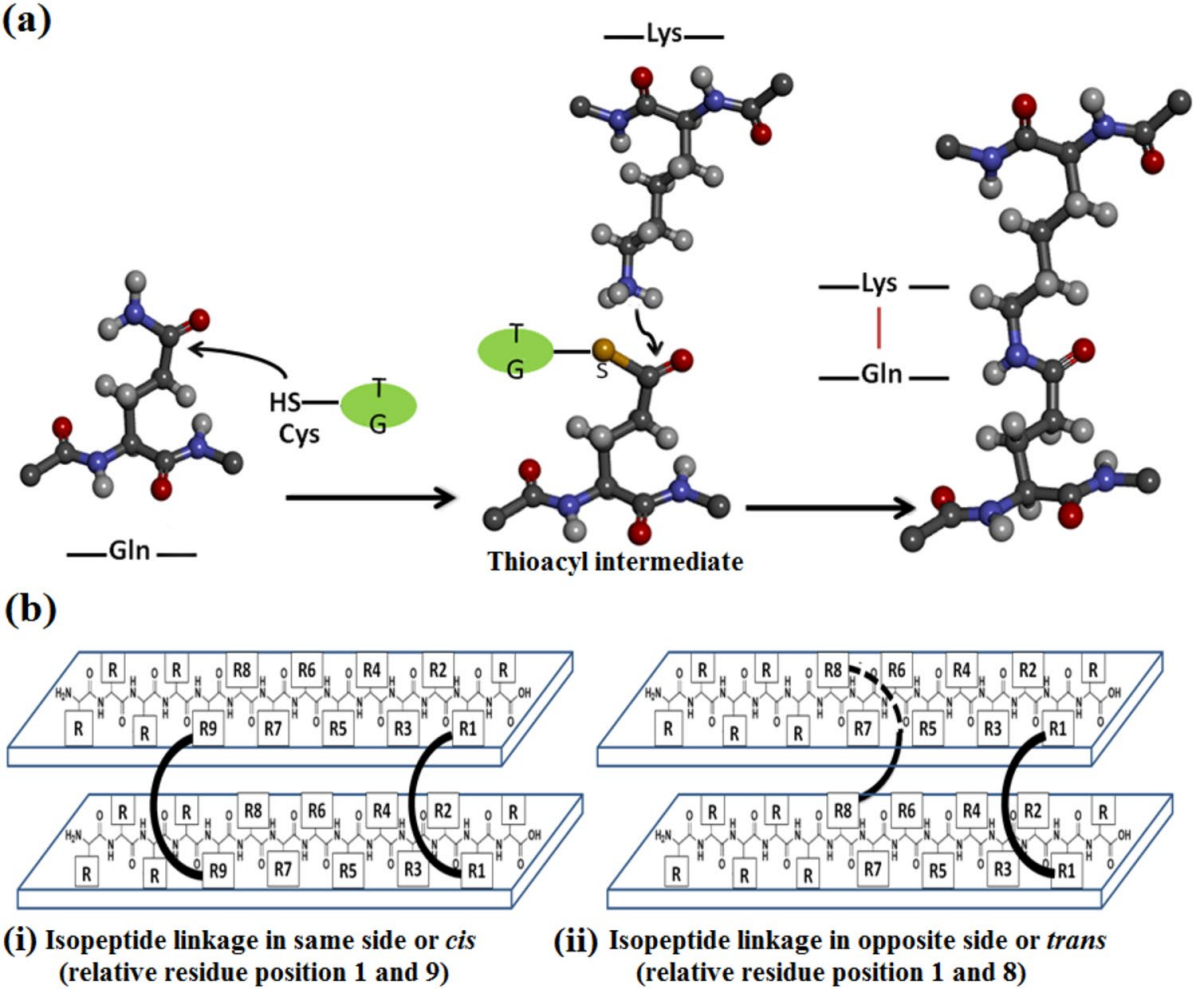
(**a**) Transglutaminase mediated formation of the intermolecular isopeptide bond. (**b**) Schematic representation of orientations of two possible isopeptide bond formation between two different peptide molecules having β-sheet conformations : (i) *cis*, where both the isopeptide bonds are formed in the same side of the two peptides and (ii) *trans*, where the isopeptide bonds are formed in the opposite side of the two peptides.

Our key focus is to design fibrin-inspired peptide based sealants, having superior hemostatic properties than fibrin. Towards this goal, we have designed 3 peptide based sealants, sealants 1–3, as described in Table 1 and Figure S1. Peptide 1 is the 14-residue segment of functional domain of human fibrinogen γ-chain at the C-terminus, “Gln-Gln-His-His-Leu-Gly-Gly-Ala-Lys-Gln-Ala-Gly-Asp-Val” and it forms sealant 1 by the self-assembly of peptide 1 in presence of TG. TG cross-links between two peptide 1 chains by forming N^ε^(γ-glutamyl) lysine isopeptide bond^4^. In natural fibrin, thrombin activated factor-XIIIa enzymatically cross-links between Lys-406 and Gln-398 or 399 on opposing C-terminal segments of the γ-chain and forms N^ε^(γ-glutamyl) lysine isopeptide bridge in presence of calcium ion^4^. Figure 1a explains transglutaminase mediated formation of intermolecular isopeptide bond. During transglutamination, a glutamine side-chain on a substrate protein or peptide is attacked by the Cys residue present in the active site of transglutaminase enzyme and forms thioacyl intermediate. Lysine side-chain amine from a second substrate molecule makes nucleophilic attack on thioacyl intermediate and forms the isopeptide bond. Figure 1b explains the orientations of two possible isopeptide bond formations between the two different peptide molecules. Based on the feasibility of dimer formation, we can subdivide the pairs in two groups: (i) *cis*, where both the isopeptide bonds are formed in the same side of the two peptides and (ii) *trans*, where the isopeptide bonds are formed in the opposite side of the two peptides in extended conformation. Sealant 2 is formed by the assembly of peptides 2 and 3 in presence of TG. Peptide 2 and 3 are having complementary charges to promote lysine-aspartate ionic interactions and designed isopeptide bond mediated interactions. Peptide 2 is Asp residue enriched 14 residue sequences having two Gln residues at 6^th^ and 13^th^ positions as isopeptide bond formation sites and peptide 3 is 14 residue sequences having all Lys residues. Incorporation of two isopeptide bond forming sites imparts additional strength and stability in the designed sealants. Similarly, sealant 3 is formed by the assembly of peptides 4 and 5 in presence of TG. Peptide 4 and 5 are having complementary charges promoting lysine-aspartate ionic interactions and designed isopeptide bond mediated interactions like sealant 2. Insertion of ^D^Ala residue in peptides 4 and 5 minimizes adjacent Lys-Lys and Asp-Asp repulsion and imparts proteolytic stability to the sealant 3. We aim to probe how the change in orientation of isopeptide bond can affect the sealant stability in case of sealants 2 and 3. Peptide 4 is 14 residue sequence, having alternate Asp and ^D^Ala residues and having two Gln residues at 4^th^ and 12^th^ positions as isopeptide bond formation sites. Peptide 5 is 14 residue sequence, having alternate Lys and ^D^Ala. TG cross-links between Gln residue of peptide 4 and Lys residue of peptide 5 and form sealant 3. In this study, we performed physicochemical characterization of peptides and sealants and also carried out *ex-vivo* functional studies to correlate structure-activity relationship.

**Table 1:** Designed sealants and peptide sequences.

Sealant code no.	Peptide code no.	Sequence
Sealant 1	Peptide 1	H-Gln-Gln-His-His-Leu-Gly-Gly-Ala-Lys-Gln-Ala-Gly-Asp-Val-OH
Sealant 2	Peptide 2	H-Asp-Asp-Asp-Asp-Asp-Gln-Asp-Asp-Asp-Asp-Asp-Asp-Gln-Asp-OH
	Peptide 3	H-Lys-Lys-Lys-Lys-Lys-Lys-Lys-Lys-Lys-Lys-Lys-Lys-Lys-Lys-OH
Sealant 3	Peptide 4	H-^D^Ala-Asp-^D^Ala-Gln-^D^Ala-Asp-^D^Ala-Asp-^D^Ala-Asp-^D^Ala-Gln-^D^Ala-Asp-OH
	Peptide 5	H-^D^Ala-Lys-^D^Ala-Lys-^D^Ala-Lys-^D^Ala-Lys-^D^Ala-Lys-^D^Ala-Lys-^D^Ala-Lys-OH

### Determination of secondary structures of designed peptides by CD spectroscopy

The secondary structures of the peptides were characterized by CD spectroscopy (Figure S8). The CD spectra of peptides 1–4 show minima at ~198 nm, inferring unstructured conformation of the peptide backbones. Such unstructured conformation is expected in short peptides, rich with polar amino acid residues^32^. Interestingly, peptide 5 shows CD spectra having two minima at 217 and 198 nm. Insertion of alternate ^D^Ala residues in peptide 5 promotes Π(LD) helical fold in peptide 5^33^, which has peptide chain oriented like a beta strand. It may be noted that in such Π(LD) helical structures alternate side-chains protrude on alternate faces of a sheet like form. The CD spectrum of fibrinogen shows helical structure with two minima at 223 and 208 nm.

### Morphological properties of fibrin-inspired sealants

**Figure 2:**
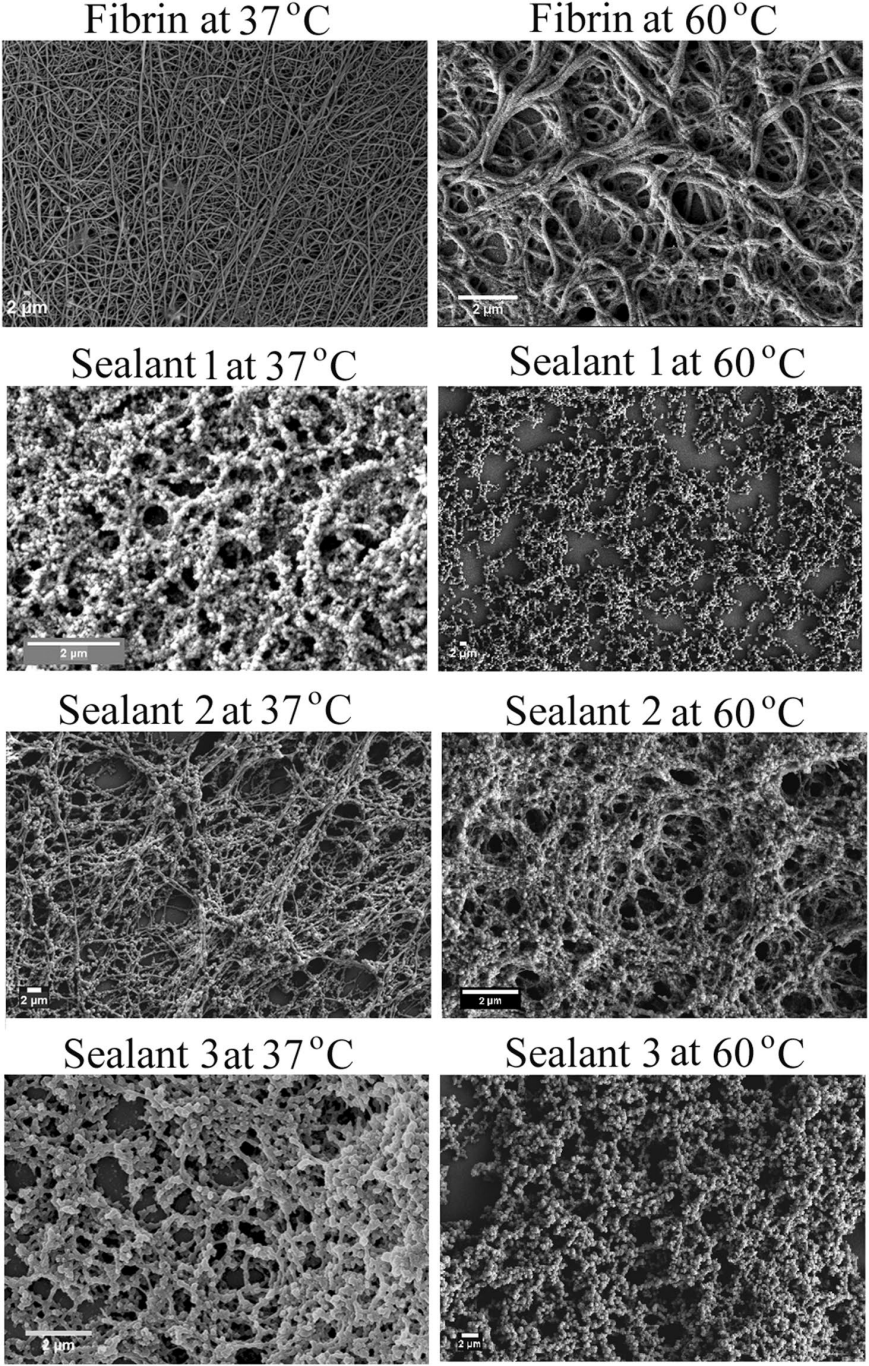
FE-SEM images of fibrin, sealant 1, 2 and 3 at 37 °C (left panel) and at 60 °C (right panel). The resolution of FE-SEM images at 37 °C of fibrin, sealant 1, 2 and 3 are 10.86, 32.7, 4.97 and 23.69 KX respectively. The resolution of FE-SEM images at 60 °C of fibrin, sealant 1, 2 and 3 are 21.06, 2.57, 19.75 and 6.05 KX, respectively. Scale bar of all the SEM images are 2 µm.

**Figure 3:**
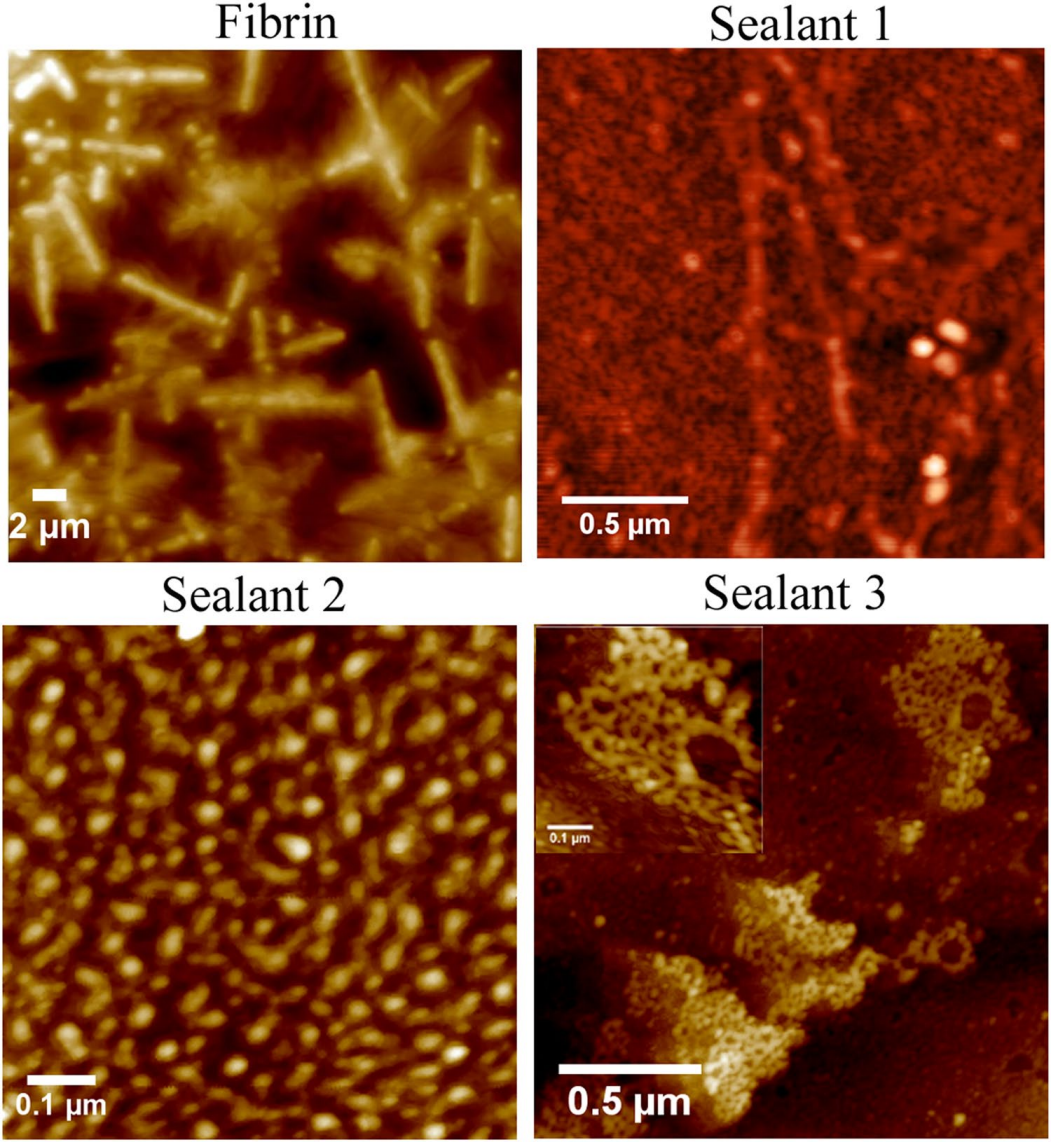
AFM images of the fibrin and sealants 1–3 taken in semi-contact mode. Scale bar of the AFM images of fibrin and sealants 1, 2 and 3 are 2, 0.5, 0.1 and 0.5 µm, respectively.

We elucidated the temperature dependent morphological properties of the designed sealants by recording FE-SEM at 37 °C and at 60 °C (Fig. 2). Sealants were prepared in presence of TG and Tris buffer having CaCl_2_, since calcium ion is needed for TG activity^4^. At 37 °C, FE-SEM images reveal fibrin forms predominantly nanofibre like morphology, whereas sealants 1, 2 and 3 show nanoparticle morphology connected by nanofibre. Probably, in sealant 2 and 3, lysine residues of peptides 3 and 5 come at the vicinity of chloride counter ion or aspartic acid residues of peptides 2 and 4 come at the vicinity of calcium counter ion and form nanoparticle like structure. Morphological analysis of SEM image of sealant 2 shows that nanoparticles are having 326 ± 27 nm diameter and width of nanobridges are 303 ± 34 nm. Nanoparticles appeared in FE-SEM image of sealant 3 are having 441 ± 27 nm diameter and the width of nanobridges are 305 ± 21 nm. Figure 2 shows that sealant 2 has retained its cross-linked structure even at higher temperature (60 °C), inferring its thermal stability. At higher temperature, the cross-linked structure of sealant 1 is disrupted and the cross-linked structures of fibrin and sealant 3 are partially disrupted. TEM images of fibrin shows fiber like morphology (Figure S9), whereas TEM images of our designed sealants (1, 2 and 3) show nanoparticle morphology connected by nanofibre (Figure S9), which directly complement the morphological structures obtained from FE-SEM analysis (Fig. 2). Semi-contact mode AFM images also reveal nano-structure pattern for the assembled peptides as shown in Fig. 3. AFM image of fibrin shows nano-fibre structure, whereas AFM images of sealants 1, 2 and 3 reveal the presence of nanoparticle like structures connected by short nanofibres. All the designed sealants are stabilized by engineered isopeptide bonds, which probably form nanobridge structures in engineered sealants.

**Figure 4:**
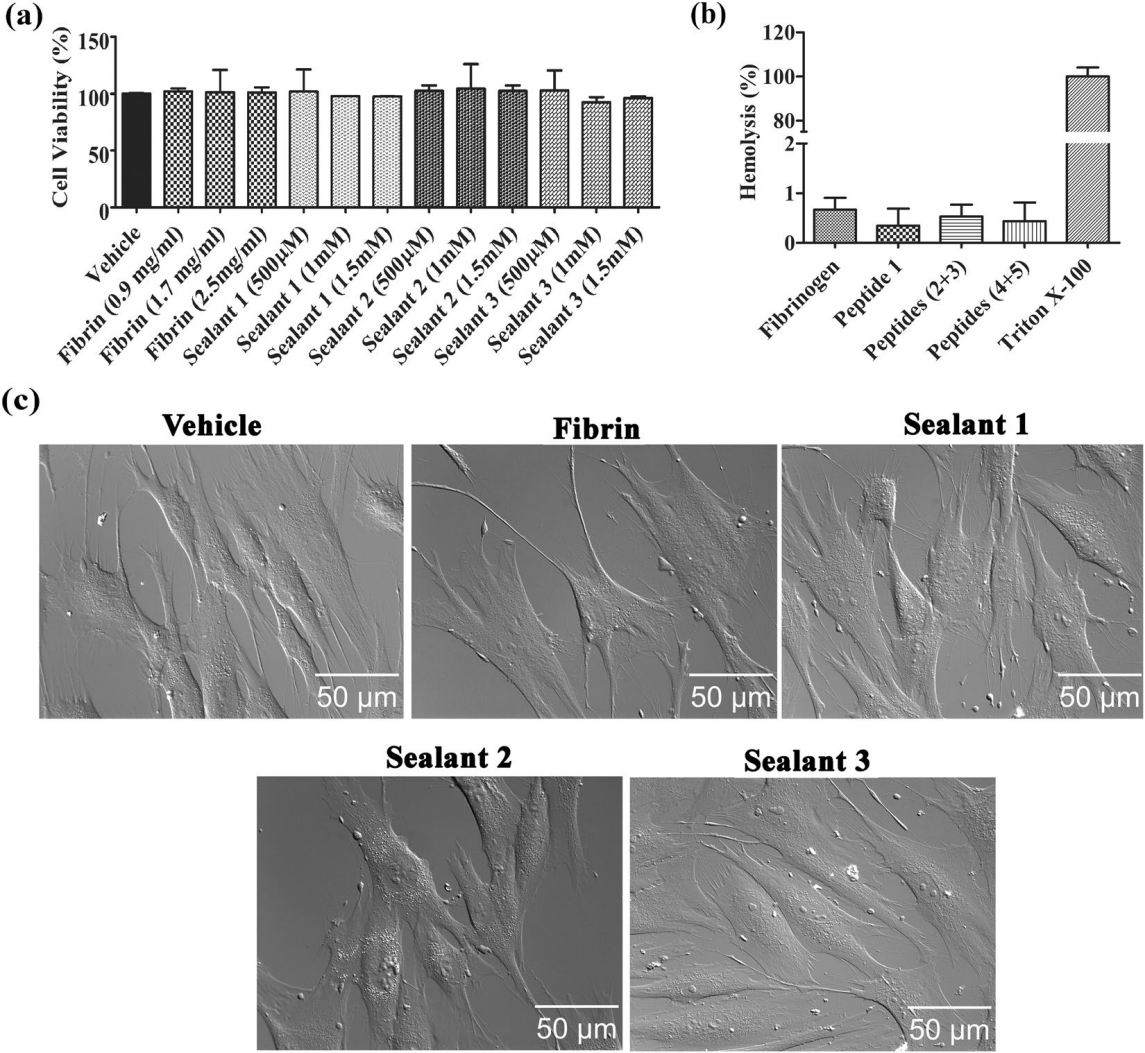
(**a**) MTT assay of sealants against human fibroblasts cells, (**b**) hemolysis assay of peptide mixtures against blood cells (chicken) and (**c**) DIC images of human fibroblast cells in presence of sealants. Scale bar of the DIC images are 50 μm.

Molecular structure and nanomechanical properties of the designed sealants are expected to play important role in blood coagulation. Blood clot needs to be stiff to stop hemorrhage, otherwise ruptured clot may cause thromboembolism^27^. We have evaluated the mechanical properties of our designed sealants using contact mode AFM (Figure S10). The cantilever behaves like a spring, thus force is proportional to deflection. Fibrin and fibrin-inspired sealant 1 have comparable strengths, having adhesive forces ~13.6 ± 1.3 and 12.3 ± 4.2 nN, respectively. Sealants 2 and 3 are having adhesion forces ~22 ± 2.7 and 18.6 ± 1.3 nN, respectively, since sealants 2 and 3 are stabilized by lysine-aspartate ionic interactions and also by N^ε^(γ-glutamyl) lysine isopeptide bond mediated covalent interaction. These form stiffer clot than fibrin. We performed cytotoxicity assay with human fibroblast cell line, examined gross morphology of fibroblast cells seeded on sealants and also checked the hemolysis assay in chicken blood cells (Fig. 4). Figure 4 shows our designed sealants are biocompatible. Sealants 2 and 3 show strong adhesive strength, stiffness, biocompatibility and hemostatic activities, which indicate that such interesting class of sealants, might be potentially used as suture less wound closure, especially, in places where it is very difficult to place suture due to absence of healthy collagen tissue.

### Computational modeling of designed sealants

**Figure 5:**
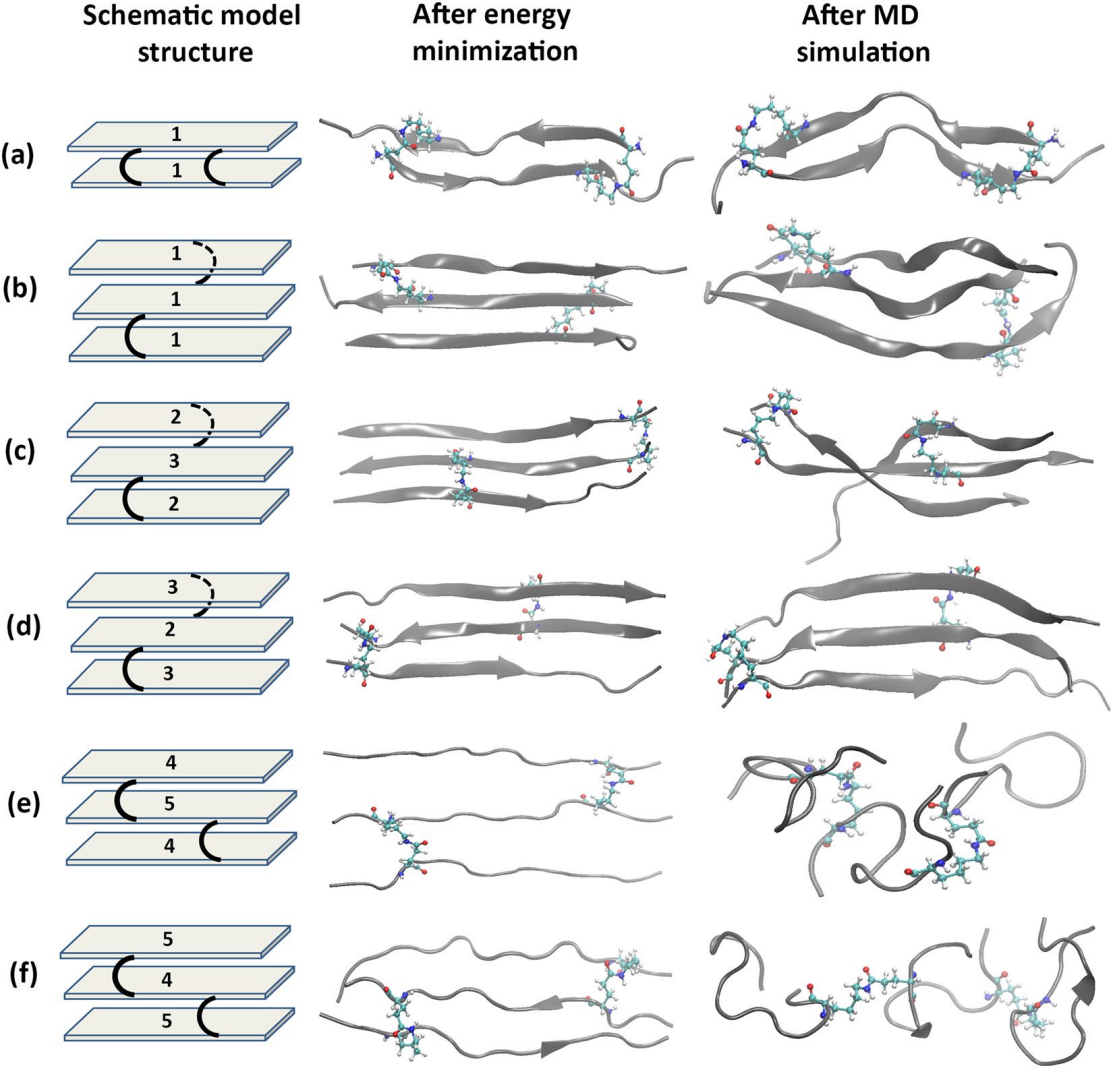
Cross-linked sealant systems: (**a**) sealant1-*cis* formed as peptide dimer, (**b**) sealant1-*trans* formed as peptide trimer, (**c**) sealant2-*trans* type1 formed as peptide trimer (two peptide2 and one peptide3), (**d**) sealant2-*trans* type2 formed as peptide trimer (two peptide3 and one peptide2), (**e**) sealant3-*cis* type1 formed as peptide trimer (two peptide4 and one peptide5) and (**f**) sealant3-*cis* type2 formed as peptide trimer (two peptide5 and one peptide1). The first column shows spatial arrangement of the isopepide cross-linking (black curved lines) and the peptides are simplified as grey bars with assigned peptide number. The second column shows energy minimized structures and the third column shows corresponding structures after MD simulation. The secondary structural elements are shown as cartoon and interacting Lys and Gln pairs forming isopeptide bonds are shown in ball and stick.

Modeling of the peptides solely reveals that formation of the isopeptide bonds may depend on some key factors. The orientation of the amino acid side-chains in space facilitates or hinders the isopeptide bond formation (Fig. 1b). After formation of the first isopeptide bond, the next one needs specific orientation in space of the interacting side-chain atoms. As the peptides carry similar charges on successive residues (Lys or Asp), it is expected that the peptide would adopt extended β-sheet conformation where side chains are projected on alternate sides as shown in Fig. 1b. The formation sites of the isopeptide linkages are predefined in natural fibrin which could be 6 or 7 amino acids apart. Considering the position of linkage formation via isopeptide bond, we can subdivide any two consecutive isopeptide bond pair in following groups: (i) *cis*, where both the isopeptide bonds are formed in the same side of the β-sheet (Figs 1b and 5a) and (ii) *trans*, where the two isopeptide bonds are in the two faces of the β-sheet (Figs 1b and 5b). Sealant 1 (Fig. 5a,b) consisting of peptide 1 can form isopeptide bonds in both *cis* and *trans* orientations. Peptide 2 and 3 forming sealant 2 (Fig. 5c,d), can form two isopeptide bonds only in *trans* orientation and peptide 4 and 5 forming sealant 3 (Fig. 5e,f), can form only *cis* isopeptide bonds in the process of generation of a peptide dimer. It could be anticipated that after formation of the first isopeptide bond, the second isopeptide bond formation in *trans* orientation will be energetically less favorable. This observation leads to explaining the extent of propagation of peptide linkage through consecutive isopeptide bond formations as shown in Fig. 5. The natural pair (peptide 1) being able to form *cis* linkages can form peptide dimer (Fig. 5a). However, if the *trans* position is triggered first, then it may lead to extension of the peptide linkage beyond dimer (Fig. 5b). Similarly, the peptides of sealant 2 is unable to form dimer as the isopeptide bonds are *trans* to each other and only extended linkage may let each peptide to form two isopeptide bonds. However, there can be two compositions of peptide combination leading to type1 and type2 trimers (Fig. 5c,d). The peptides in sealant 3 can potentially form dimer only as the two isopeptide bonds are *cis*, but insertion of D-amino acid as spacer residue in sealant 3 leads to steric crowding. Hence it is anticipated to form elongation if proper orientation is achieved. Here also, the elongated trimer can form two different peptide combination (Fig. 5e,f) leading to type1 and type2.

**Figure 6:**
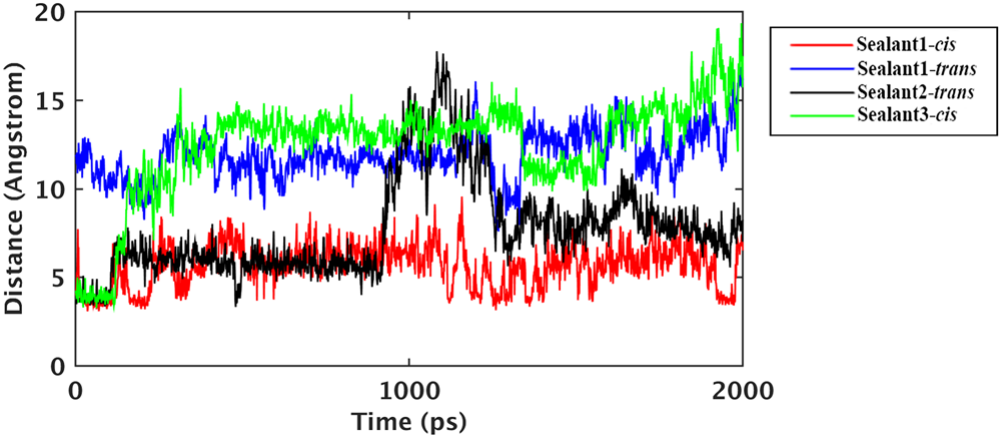
Distance between N^ε^ of Lys and C^δ^ of Gln of the second isopeptide bond forming site for the dimers having following special arrangements: sealant 1-*cis* (red), sealant 1-*trans* (blue), sealant 2-*trans* (black) and sealant 3-*cis* (green) for 2ns simulation, having one prior isopeptide linkage.

In order to get a quantitative measurement of the proposed theory, we ran 4 short MD simulations, where, one isopeptide bond is formed (Table S2) and the distance (d_iso_) between second isopeptide bond forming atoms (N^ε^ of Lys and C^δ^ of Gln) is monitored (Fig. 6). In sealant 1, the initial isopeptide bond formation, which will trigger the next isopeptide bond in *cis* position, has mean d_iso_ of 5.62(±1.20) Å. The same sealant with second isopeptide bond forming site at *trans* with respect to β-sheet, shows mean d_iso_ of 11.94(±1.34) Å. Hence, the *cis* arrangement will lead to the dimer formation, while the *trans* arrangement may facilitate elongation through peptide polymerization. Similarly, in sealant 2, where initial isopeptide bond formation triggers the next isopeptide bond in *trans* position has mean d_iso_ of 7.68(±2.77) Å. Hence, in this case, the polymerization is probably more feasible than dimer formation. This structure also shows much higher fluctuation of d_iso_. For sealant 3, where primarily formed isopeptide bond triggers the second isopeptide bond in *cis* position has mean d_iso_ of 12.49(±2.28) Å. The large d_iso_ in case of *cis* configuration in sealant 3, indicates formation of unstructured peptide assembly. Again, the d_iso_ in sealant 3 shows high fluctuation which may limit its degree of assembly formation. The time evolutions of the distances (d_iso_) are shown in Fig. 6.

### Molecular Dynamics (MD) simulations of designed sealants

All atom MD simulations in water bath were done on the six focused structures generated from the above mentioned observations gained from initial modeling and distance monitoring as shown in Fig. 5 schematic model structures. The schematic models show that the dimer (sealant 1) is formed by two isopeptide bonds between two peptides, whereas the trimer is formed by two isopeptide bonds between three peptides (sealants 1–3). Structural fluctuations of the cross-linked sealants are evaluated by time evolution plot of root-mean-square-deviation (RMSD) with respect to the energy minimized structures (Figure S11a). Sealant 1-*cis* and sealant 1-*trans* show similar RMSD values. However, the sealant 2-*trans* type2 formed by assembly of three peptides depicts less RMSD fluctuation relative to all other systems. Sealant 3-*cis* type1 and type2 formed by three cross-linked peptides containing D-Ala residue as spacer, shows highest fluctuation which is evident from the lack of definite structural pattern of the peptides even at the stage of energy minimization and throughout the simulation (Fig. 5e,f). The radius of gyration (R_g_), which is the parameter for the spread of the molecule from its centre, is calculated as the time evolution of R_g_ (Figure S11b). Sealant 2-*trans* type2 shows highest and stable R_g_ value, which indicates it has least deformed or contracted among other structures. Nevertheless, very less fluctuation in R_g_ of both sealant 2-*trans* type1 and type2 is associated with lesser change in structure during simulation. Both sealant 3-*cis* type1 and type2 show higher fluctuation in R_g_. The root-mean- square fluctuations (RMSF) of individual residues (Figure S12) also show higher fluctuation for sealant 3-*cis* for both type1 and type2.

Solvent accessible surface areas (SASA) have been calculated which shows that the time evolution of SASA depicts no major change in the surface area (Figure S13), however, higher fluctuation in surface area is observed in sealant 3-*cis* for both type1 and type2. The secondary structural information reveals that some of the sealants retained or acquired partial β-sheet conformations (Figure S14). The percentage of β-sheet character is highest for sealant 2-*trans* for both type1 and type2 (nearly 40%), while it is lowest or almost unavailable for the two sealant 3-*cis* assemblies. It may be mentioned that TEM image (Figure S9) of sealant 2 shows fibrous kind of structure, which supports the partial β-sheet conformation of the simulated structures^34^. The internal rigidity can be explained by hydrogen bond interaction within the cross-linked sealant structures. To compare the structures, a parameter is defined by average lifetime in picoseconds of all hydrogen bonds (acceptor-donor hydrogen bond distance cutoff of 2.4 Å and angle cut off of 150°) within the peptide residues divided by the number of residues in that particular structure, denoted by h_pep_. The h_pep_ values are 0.16, 0.12, 0.19, 0.16, 0.12 and 0.13 hydrogen bonds/residue for sealant 1-*cis*, sealant 1-*trans*, sealant 2-*trans* type1, sealant 2-*trans* type2, sealant 3-*cis* type1 and sealant 3-*cis* type2 respectively, whereas, h_solv_ (hydrogen bond between peptide and solvent divided by residue number) are 0.14, 0.09, 0.11, 0.11, 0.10 and 0.10 respectively. The h_pep_ values indicate that sealant 2-*trans* type1 and type2 both contain most internal hydrogen bonds among others, owing to its diminished structural fluctuation and β-sheet like structural features. The h_solv_ parameter shows that sealant 1-*cis* have higher interaction with the solvent through hydrogen bonding and all other assemblies have similar hydrogen bonding with water, indicating almost similar solubility.

### Isopeptide bond stabilized sealant peptide clotting mechanism and determination of cross-linking sites in sealants

**Figure 7:**
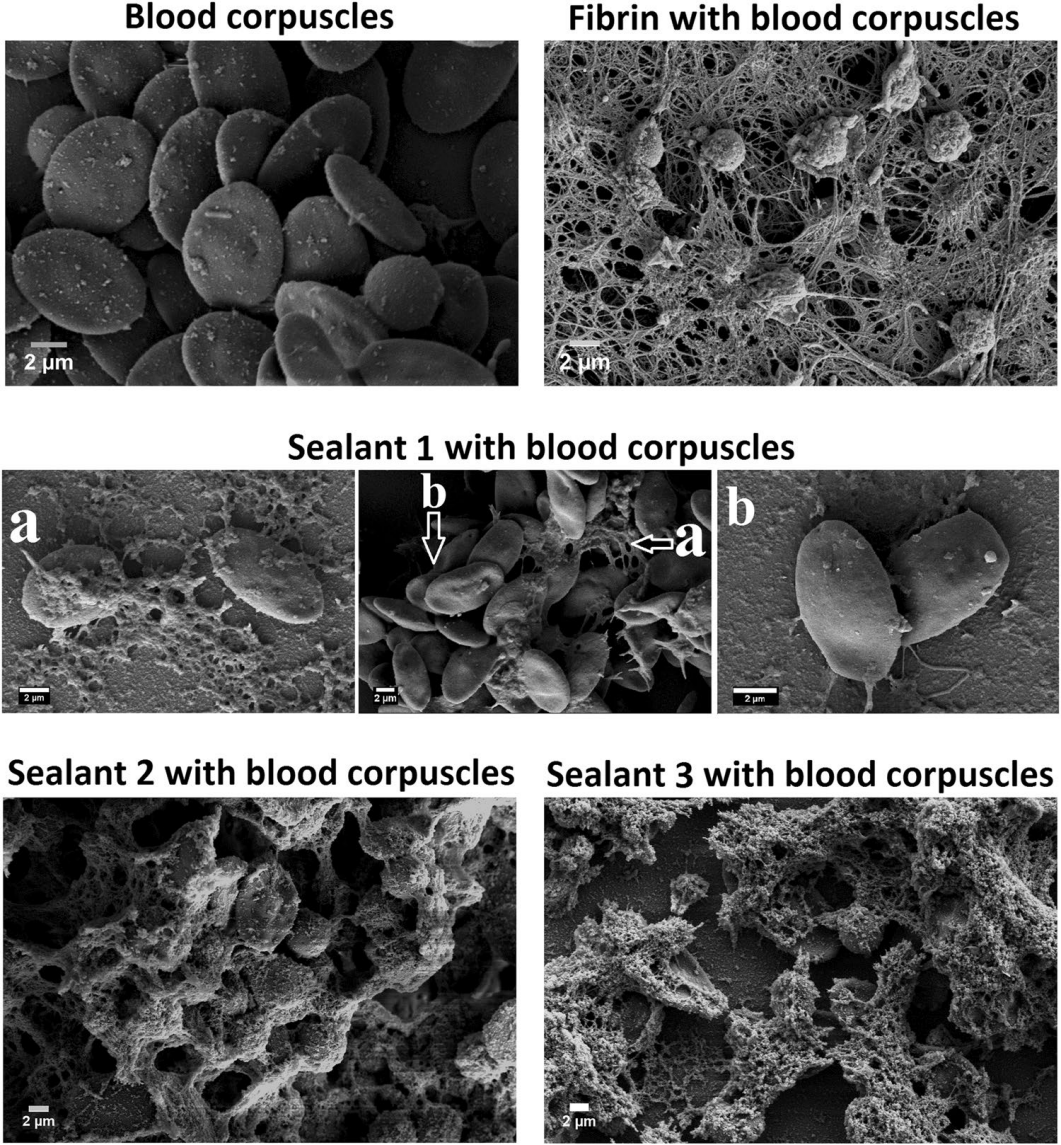
SEM images of indigenous fibrin free blood corpuscles and blood corpuscles with fibrin and sealants. SEM image (resolution 7.79 KX) of sealant 1 with blood corpuscles shows (**a**) blood corpuscles loosely entrapped by sealant 1 and (**b**) blood corpuscles not entrapped by sealant 1. The resolution of SEM images of indigenous fibrin free blood corpuscles and blood corpuscles with fibrin, sealant1(middle), sealant 2 and sealant 3 are 9.34, 9.01, 11.32, 5.99 and 5.82 KX, respectively. Scale bar of the SEM images are 2 μm.

The process of blood clotting starts when fibrinogen is activated by thrombin to form nanofibrous structures to entrap blood corpuscles and platelet. The self-assembling peptides have ability to coagulate blood by forming nanofiber to entrap blood corpuscles^14^. We examined the formation of blood clot by designed sealants using scanning electron microscopy (Fig. 7). To examine the mechanism of hemostasis of sealants 1–3 and fibrin in a wound, we mimicked the scenario *ex-vivo* by combining fibrin and sealants 1–3 with blood corpuscles. FE-SEM images reveal that fibrin and sealants 2 and 3 form interwoven nanofiber with visibly entrapped blood corpuscles (Fig. 7). Morphological examination of FE-SEM images infer that sealant 2 and 3 nanofibres interact with blood corpuscles in a similar fashion like fibrin. Sealant 1 is made of functional segment of fibrinogen, but it predominantly fails to entrap the blood corpuscles as evident from Fig. 7, but in certain places sealant 1 entrapped blood corpuscles are also observed. We were interested to know the mechanism of TG mediated cross-linking in designed sealants responsible for entrapping blood corpuscles. Integrating blood corpuscles entrapped SEM images data and computational studies, we infer two isopeptide bonds between the two peptide fragments, forming a peptide dimer, may not facilitate in entrapping the blood corpuscles. For sealants 1–3, trimer and higher oligomers having one isopeptide bond between two peptide fragments probably facilitate in entrapping the blood corpuscles (Figs 5 and 7, Figures S15–17). Interestingly, in natural fibrin two isopeptide bonds formation occur between two fibrinogen proteins and the functional segment of fibrinogen adopt coiled coil structure^35, 36^. Since, the formation of nanofiber entanglements occur under anti-coagulating condition, our designed sealants 2 and 3 may exhibit significant hemostatic activity even under coagulopathic conditions. Figure S18 shows the *ex-vivo* clotting time determination of sealants with blood corpuscles in PBS by Hayem method^37^. Fibrin, sealant 1, 2 and 3 entrap blood corpuscles in ~56 ± 2, 51 ± 5.3, 21.3 ± 1.5 and 26.3 ± 1.5 secs, respectively. Blood-clotting experiment by Hayem method was performed to compare the efficacy of designed sealants in absence of indigenous fibrin. We also performed thrombin clotting time experiment by incubating platelet-free plasma and the period of clot formation was observed (Figure S19)^38^. The clotting time in presence of thrombin, sealants 1, 2 and 3 are ~32 ± 2, 35 ± 1, 25 ± 1 and 28 ± 2 secs, respectively. Thrombin clotting time experiment was performed to demonstrate the plasma clotting efficiency of our designed sealants like fibrin. In our designed sealants, we have introduced two cross-linking sites for forming efficient sealants for blood-clotting. Due to two cross-linking sites, in the designed sealants, the sealants formed large heterogeneous polymerized structures having limited solubility, which prevented their accurate mass determination. For mass spectroscopic characterization of N^ε^-(γ-glutamyl)-lysine isopeptide bond formation in our sealants 1–3, we synthesized two model tetrapeptides, H-Ala-Lys-Ala-Val-OH and H-Ala-Gln-His-Val-OH, having one cross-linking site and performed TG-mediated isopeptide bond formation. LC-ESI-MS/MS studies support the formation N^ε^-(γ-glutamyl)-lysine isopeptide bond in cross-linked octapeptide and also in daughter ions arising from the cross-linked peptide (Figures S22–S26).

In this report, our designed sealants 2 and 3 facilitate superior hemostasis compared to fibrin and form fibrin-like nanofibre entanglements to entrap blood corpuscles. Computational studies reveal that the elongation of cross-linked peptide assembly depends on the relative position of the interacting side chains and isopeptide bond formation sites, which can guide future design of improved sealants having superior proteolytic and thermal stability. In conclusion, our engineered sealants have demonstrated superior mechanical and hemostatic properties than fibrin and such sealants might be potentially translated to clinics for treating impaired blood clotting, traumatic coagulopathy and even for suture free wound closing.

## Methods

### Materials

All Fmoc protected amino acids were purchased from GL Biochem and resins were purchased from Novabiochem and used without further purification. The coupling reagent 2-(1H-benzotriazol-1-yl)-1,1,3,3-tetramethyluronium hexafluorophosphate(HBTU) and 1-[bis(dimethylamino)methylene]-1H-1,2,3-triazolo[4,5-b]pyridinium3-oxide hexafluorophosphate (HATU), were purchased from Novabiochem and hydroxybenzotriazole (HOBt) was purchased from SRL (Sisco Research laboratory). The anhydrous dimethylformamide (DMF) and dichloromethane (DCM) were purchased from Acros Organics and anhydrous N,N-diisopropylethylamine (DIPEA), hexamethyldisilazane (HMDS), transglutaminase (guinea pig liver), fibrinogen (human plasma﻿), thrombin (bovine plasma) and 2,4,6-trinitrobenzenesulfonic acid (TNBS) were obtained from Sigma-Aldrich. HPLC grade methanol and ethanol were procured from Merck-Milli pore. All procured chemicals are certified to be ~98–99% purity.

### Synthesis of sealant peptides

All peptides were synthesized by employing Fmoc solid phase synthesis strategy (Figures S1 and S2). Fmoc-Val-Wang resin (100–200 mesh, NovaBiochem), Fmoc-Asp-Wang resin (100–200 mesh, NovaBiochem) and Fmoc-Lys-Wang resin (LL, 100–200 mesh, Novabiochem) having loading capacity 0.1 mmol/g were used. Each coupling reaction was employed with 5 equivalent of Fmoc protected amino acid, 4.8 equivalent of HBTU and 5 equivalent of HOBt with 10 equivalent of diisopropylethylamine (DIPEA) and was allowed to progress at room temperature for an hour (Figure S2). For Lys-^D^Ala or Asp-^D^Ala coupling reaction, reaction time was extended upto 3 hours using HATU as coupling reagent. Each coupling reaction completion was monitored by TNBS test for primary amine. After each coupling, Fmoc group was removed by 20% (v/v) piperidine in DMF. To prevent aspartimide formation for Asp-Gln-Asp segment, we used Fmoc-Asp(OMpe)-OH (GL Biochem), -derivative instead of Fmoc-Asp(OtBu)-OH^39^. The resin was washed with DMF, DCM and MeOH and dried under high vacuum before de-protection. The de-protection of peptide from resin was carried out by treatment with 50:45:2.5:2.5 (v/v) (TFA: DCM: TIS: water) for 3 hours. The resin was filtered off and the filtrate was treated with cold ether and the peptide was separated by centrifugation. The peptide was purified using HPLC on a C18 column. The each peptide was confirmed by MALDI-TOF mass spectra (Table S1 and Figures S3–S7).

### Circular Dichroism (CD) studies of peptides

CD was recorded on a JASCO spectropolarimeter using a cell of 1 mm path length. Peptides were dissolved in 50 mM Tris HCl buffer having pH 7.4 and peptide concentrations of 250 μM for peptide 2–5, 300 μM for peptide 1 and 3 μM for fibrinogen. CD spectra were recorded in the range of 195–280 nm wavelengths. CD spectra were obtained as accumulation of three scans using a scan speed of 100 nm/min, data pitch of 0.2 nm and band width 1.0 nm. Each spectrum was subtracted from the buffer and smoothened and plotted using origin pro 8 (Figure S8).

### Transglutaminase mediated cross-linking and preparation of sealant solution for TEM, FE-SEM and AFM studies

6 mM peptide stock solutions were prepared in milli-Q water. For microscopic studies for sealant 1, 1 μL of peptide 1 and 1 μL of milli-Q water were mixed. Similarly, for sealant 2, 1 μL of peptide 2 and 1 μL of peptide 3 were mixed and for sealant 3, 1 μL of peptide 4 and 1 μL of peptide 5 were mixed and incubated at room temperature for 8 hrs. 4 μL of buffer (50 mM Tris HCl + 2.5 mM CaCl_2_, pH 7.4) and 1 μL of TG (7 U/mL) were added to form each sealant and the mixtures were incubated for 30 minutes at 37 °C for TEM, FE-SEM and AFM studies and for checking the thermal stability of sealants by FE-SEM, the sealant samples and fibrin solution were also incubated separately at 60 °C. 1 μL of 15 mg/mL fibrinogen and 1 μL of milli-Q water, 4 μL of buffer (50 mM Tris HCl + 2.5 mM CaCl﻿_2_﻿, pH 7.4) and 1 μL of thrombin (10 U/mL) were mixed to prepare fibrin solution.

### Transmission Electron Microscopy (TEM) images of sealants

5 µL of sealant solution was drop casted on a freshly glow-discharged carbon coated 300 mesh copper grid and incubated for 5 min at room temperature. The excess solution was wiped out and washed with water and then stained with 2% (w/v) uranyl acetate. The morphology of sealants were characterized once by using Transmission Electron Microscope (Tecnai G2-F20 ST, FEI Company) with accelerated voltage of 120 kV and 200 kV (Figure S9).

### Field Emission Scanning Electron Microscopy (FE-SEM) images of sealants

All sealant solutions were fixed with 2% (v/v) glutaraldehyde in 25 mM PBS at pH 7.4 for overnight at 4 °C. A drop of sample was placed on a glass cover slip and allowed to semi-dry in air. The specimens were placed into 2:1, 1:1 and 1:2 (v/v) solutions of EtOH-Hexamethyldisilazane (HMDS, Sigma) mixtures, followed by three times treatment with 100% HMDS and allowed to air dry. HMDS is considered as a good alternative of critical point drying^15, 26^. The specimens were then mounted onto FE-SEM stubs affixed with double-stick conductive carbon tape and sputter coated with gold-palladium and were analyzed using SUPRA 55VP-Field Emission Scanning Electron Microscope (Zeiss company) (Fig. 2). This instrument has high performance variable pressure FE-SEM with patented GEMINI column technology, Schottky type field emitter system, single condenser with crossover-free beam path, resolution: 1.0 nm at 15 kV; 1.6 nm at 1 kV high vacuum mode and 2.0 nm at 30 kV at variable pressure mode. We have repeated this experiment twice.

### FE-SEM images of sealants with blood corpuscles

Serum free blood corpuscles were prepared by centrifugation of citrated whole chicken blood and stored at 4 °C. Citrated serum free blood (5 μL) was quickly mixed with each sealant separately and spotted on glass cover slip. The clot was allowed to set for 30 minutes at 37 °C, followed by fixing with 2% (v/v) glutaraldehyde for overnight at 4 °C. After fixation, samples were rinsed with Tris HCl and dehydrated. The specimens were placed into 2:1, 1:1 and 1:2 (v/v) solutions of EtOH-HMDS mixtures, followed by three times treatment with 100% HMDS and allowed to air dry. The specimens were then mounted onto FE-SEM stubs affixed with double-stick conductive carbon tape and sputter coated with gold-palladium. The samples on glass were coated using gold−palladium and were analysed using SUPRA 55VP-field emission scanning electron microscope (Zeiss Company) (Fig. 7). We have repeated this experiment twice.

### Atomic Force Microscopy (AFM) studies of sealants

AFM images of sealants (Fig. 3) and adhesion force (Figure S10) of sealants were measured by VEECO AFM instruments. Sealant solutions were deposited onto gold sputtered cover slips and allowed to adsorb for 5 min. Silicon nitride probes (Force constant 0.06 N/m, BRUKER SNL-10) were used for force measurement. This measurement was repeated twice. The spring constant of the cantilever was calibrated with the automated thermal fluctuation method, resulting in a value of 0.06 N/m–0.097 N/m. Molecules were stretched by first pressing the cantilevers on the gold-coated cover slips for 1 to 3 s at 500 pN to 1 nN^40–42^. The pulling speed ranged from 400 nm/s to 1000 nm/s. Gold-coated cover slips were used because they resulted in a better success rate than glass cover slips even in the absence of thio-gold bonds. We have repeated this experiment twice.

### Cell culture

Human fibroblast cells were cultured in Dulbecco’s modified Eagle medium (DMEM, purchased from Invitrogen) with 10% Bovine serum (Gibco) and 1% penicillin-streptomycin.

### Cell viability assay

To study the toxicity of the sealant peptides cell viability assay (MTT assay) was carried out (Fig. 4a)^43^. 40 μL of the fibrin (having fibrinogen concentrations 0.9 mg/mL, 1.7 mg/mL and 2.5 mg/mL) and sealant solutions with different concentrations (500 µM, 1 mM and 1.5 mM) were added into a 96-well plate and incubated for 12 h at 37 °C to form the sealant. Human fibroblast cells were seeded (10,000 cells/well) to fibrin and designed sealants in 96-well plates and incubated for 48 h in phenol-red free DMEM. Each condition was taken in triplicates. After 48 h, 10 μL of 5 mg/mL MTT solution (3-(4,5-dimethylthiazol-2-yl)-2,5-diphenyltetrazolium bromide) was added in each well of the 96-well plate and incubated for 3 h. Then 150 μL of DMSO was added to dissolve the formazan crystal and the absorbance was measured at 570 nm at UV-Vis plate reader (Molecular Devices Spectramax 190). MTT data was plotted using GraphPad Prism, and values are represented as mean ± SD of three independent experiments (Fig. 4a)^43^.

The morphology of human fibroblast cells on fibrin and the designed sealant surface were observed under apotome microscopy (Fig. 4c). In coverglass bottom disk, 60 µL of fibrin (2.5 mg/ml) and sealants (1.5 mM) were added and incubated at 37 °C for 12 h. Human fibroblast cells (5000 cells/well) were seeded on the sealant surface and incubated for 2 h in a 37 °C incubator and DIC images of unfixed cells were taken after 24 h under apotome microscope^43^.

### Hemolysis assay

Plasma free blood corpuscles were prepared by centrifugation of citrated whole chicken blood and washed three times with PBS at 4000 rpm, for 10 min at 4 °C. 50 μL of blood corpuscles were suspended in PBS at 10% (v/v) were mixed with sealant solutions (without TG) with final conc. of 1 mM in an autoclaved 0.5 mL microcentrifuge tube and incubated for 1 h at 37 °C. After incubation, each microcentrifuge tube was centrifuged at 4000 rpm for 10 min, and absorbance of the supernatants was measured at 540 nm to check hemoglobin release using Molecular Devices (Spectra Max 190) UV-Vis plate reader. Hemoglobin release in PBS and in 0.1% (v/v) Triton X-100 were used as negative (0% release) and positive control (100% release) respectively (Fig. 4b)^43^.

### Determination of clotting time

Serum free blood corpuscles were prepared by centrifugation of the citrated whole chicken blood and stored at 4 °C. Peptides were dissolved in 25 mM PBS buffer, pH 7.4. In pelleted blood corpuscles, 1 mL of 25 mM PBS, pH 7.4 was added. In 0.5 mL microcentrifuge tube, 10 μL of TG (7 U/mL), 20 μL of each peptide solution and 100 μL of the PBS solution having blood corpuscles were mixed for clotting experiment and microcentrifuge tube was slightly tilted in every 5 sec to check clot formation and clotting time was noted (Figure S18). The final concentration of each peptide was 1.5 mM (2.5 mg/mL) in the sealant. This procedure was repeated twice^37^.

### Determination of clotting time by Thrombin clotting time experiment

Plasma was collected by centrifuging whole non-coagulated chicken blood (4500 rpm, 10 min, 4 °C). Thrombin clotting time (Figure S19) experiment was performed by incubating 0.35 mL platelet-free plasma, in a 1.5 mL microcentrifuge tube at 37 °C for 10 min, followed by the addition of the thrombin-calcium reagent, 0.15 mL, maintained at room temperature^38^. The period of clot formation was noted. The same batch of platelet-free plasma was used to determine sealant clotting time. Platelet-free plasma 0.35 mL, was taken in a 1.5 mL microcentrifuge tube incubated at 37 °C followed by addition of 0.15 mL sealant solution. Sealant was prepared by dissolving each peptide in 25 mM PBS buffer, pH 7.4 with 5 mM CaCl_2_ where final conc. of the peptide was 1.5 mM followed by the addition of 10 µL TG (7 U/1 mL) soln. The period of clot formation was noted^38^. The results are represented as mean ± SD of two independent experiments.

### LC-ESI-MS/MS sample preparation for indentifying Lys-Gln cross-linked model peptide

10 mM of each peptide stock solution in milli-Q water was taken. 5 μL of peptide H-Ala-Lys-Ala-Val-OH (Figure S20) and 5 μL of H-Ala-Gln-His-Val-OH (Figure S21), were mixed with 10 μL of 25 mM Tris HCl buffer at pH 7.4. 4 μL of 10 U/mL TG in milli-Q water was first mixed with 1 μL of 10 mM CaCl_2_ for activating the enzyme, and the mixture (5 μL) was then immediately added into 20 μL of mixed model peptides solution, followed by incubation at 37 °C for 8–12 h. After TG induced cross-linking formed in the model peptide, the solution was treated with 5 mM EDTA for calcium removal followed by desalting with ZipTip C18 (tipsize P10, millipore, ZTC18S008). After being lyophilized to dryness, the desalted cross-linked model peptide was resuspended in 50% acetonitrile for LC-ESI-MS/MS analysis (Bruker HCT ULTRA ETD II, at proteomics facility, Molecular Biophysics Unit, IISc Bangalore) (Figures S22–S26).

### MD study of the sealant pairs

All atom MD simulations were done on the model structures in explicit water medium to find the structural orientation of the isopeptide bonded peptides (Table 1). Short MD simulations (2ns) were performed for four different systems comprised of pairs of peptides with only one isopeptide cross-linking bond: (i) sealant 1 peptide pair with isopeptide bond at orientation 1(ii) sealant 1 peptide pair with isopeptide bond at orientation 2 (iii) sealant 2 peptide pair and (iv) sealant 3 peptide pair (Table S2). Another set of six MD simulations were performed for 10ns each for the possible clusters of peptides with all possible isopeptide bonds, the systems are, (i) two interacting sealant 1 peptides forming two isopeptide (*cis*) linkages (sealant 1-*cis*) (ii) three interacting sealant 1 peptides forming two isopeptide (*trans*) linkages (sealant 1-*trans*) (iii) three interacting peptides (2 units of peptide 2 and 1 units of peptide 3) forming sealant 2 with two isopeptide (*trans*) linkages (sealant 2-*trans* type1) (iv) three interacting peptides (1 unit of peptide 2 and 2 units of peptide 3) forming sealant 2 with two isopeptide (*trans*) linkages (sealant 2-*trans* type2) (v) three interacting peptides (2 units of peptide 4 and 1 unit of peptide 5) forming sealant 3 with two isopeptide (*cis*) linkages (sealant 3-*cis* type1) and (vi) three interacting peptides (1 unit of peptide 4 and 2 units of peptide 5) forming sealant 3 with two isopeptide (*cis*) linkages (sealant 3-*cis* type2). The *cis*/*trans* orientation is defined by the relative positions of any two isopeptide bonds with respect to extended β-sheet like propagation. The initial models were generated as extended conformations and the isopeptide bonds were formed through CHARMM software^44^ using CHARMM36 force-field^45, 46^. A patch residue was created for isopeptide bond formation (List S1) using CHARMM36 force-field analogy. All the four systems were solvated by cubic TIP3P water boxes, which extended at least 10Å away from any peptide atom. For all four cases, the overall charges of the systems were neutralized by adding appropriate number of Na^+^ and Cl^−^ counter ions. The systems were first minimized to eliminate the initial stress and then equilibrated under the constant pressure (P = 1 atm), and constant temperature (300K) using NAMD software^47, 48^. All the simulations were carried out with 2 fs time step with SHAKE^49^ implementation in NAMD and the trajectories stored every 1 ps snapshots for the analysis. The Particle Mesh Ewald method^50^ was used to calculate the long-range electrostatic interaction. Periodic boundary conditions and a 10 Å cut-off were applied for real space non-bonded interactions.

## Electronic supplementary material

Supplementary Information
