# Supplementary material for: Engineered isopeptide bond stabilized fibrin inspired nanoscale peptide based sealants for efficient blood clotting
Source: Sci Rep. 2017 Jul 26;7:6509. doi: 10.1038/s41598-017-06360-3 (PMC5529531; doi:10.1038/s41598-017-06360-3)
Supplement: Supplementary file 1 — Supplementary Information [file 41598_2017_6360_MOESM1_ESM.doc]

Supporting Information

Engineered isopeptide bond stabilized fibrin inspired nanoscale peptide based sealants for efficient blood clotting.

Snehasish Ghosh^1^, Sanchita Mukherjee^2^, Chiranjit Dutta^1^, Kasturee Chakraborty,^2^ Paramita Gayen^2^, Somnath Jan^2^, Dhananjay Bhattacharyya^3*^ and Rituparna Sinha Roy^2*^

^1^Department of Chemical Sciences, Indian Institute of Science Education and Research Kolkata, Mohanpur – 741246, West Bengal, India.

^2^Department of Biological Sciences, Indian Institute of Science Education and Research Kolkata, Mohanpur – 741246, West Bengal, India.

^3^Computational Science Division, Saha Institute of Nuclear Physics, Kolkata, 1/AF Bidhannagar, Kolkata 700064, India.

Corresponding authors: [rituparna@iiserkol.ac.in](mailto:rituparna@iiserkol.ac.in) (experimental part) ; [dhananjay.bhattacharyya@saha.ac.in](mailto:dhananjay.bhattacharyya@saha.ac.in) (theoretical part)

**Table of Contents**

1. Figure S1. Schematic diagram of peptides 1-5.
2. Figure S2. General scheme of peptide synthesis.
3. Table S1. Peptide sequences and their mass observed in MALDI.
4. Figure S3. MALDI mass spectrum of peptide 1.
5. Figure S4. MALDI mass spectrum of peptide 2.
6. Figure S5. MALDI mass spectrum of peptide 3.
7. Figure S6. MALDI mass spectrum of peptide 4.
8. Figure S7. MALDI mass spectrum of peptide 5.
9. Figure S8. CD spectra of fibrinogen and peptides 1-5 in 50 mM Tris HCl, pH 7.4

buffer.

1. Figure S9. TEM images of fibrin, sealants 1, 2 and 3.
2. Figure S10. Force–distance curves for fibrin, sealants 1, 2 and 3 showing attraction

and repulsion using silicon nitride tip.

12. Figure S11. Measurement of structural fluctuation : (a) Root-Mean-Square Deviation (Å) and (b) Radius of Gyration (Å) of the sealant 1-*cis* in dimer form (red), sealant 1-*trans* in trimer form (blue), sealant 2-*trans* in trimer type1 form (black), sealant 2-*trans* in trimer type2 form (green), sealant 3-*cis* in trimer type1 form (cyan) and sealant 3-*cis* in trimer type2 form (magenta) on simulation data.

1. Figure S12. Root-Mean-Square Fluctuation (RMSF) (Å) of individual residues in all peptides taking into consideration only backbone atoms of the residues for sealant 1-*cis* in dimer form (red), sealant 1-*trans* in trimer form (blue), sealant 2-*trans* in trimer type1 form (black), sealant 2-*trans* in trimer type2 form (green), sealant 3-*cis* in trimer type1 form (cyan) and sealant 3-*cis* in trimer type2 form (magenta) on simulation data.
2. Figure S13. Time evolution plot of Solvent Accessible Surface Area (SASA) in Å^2^ for each sealant. For sealant pair 1 in dimer form (red), sealant pair 1 in trimer form (blue), sealant 2-*trans* in trimer type1 form (black), sealant 2-*trans* in trimer type2 form (green), sealant 3-*cis* in trimer type1 form (cyan) and sealant 3-*cis* in trimer type2 form (magenta) on simulation data.
3. Figure S14. Time evolution plot of secondary structural element: percentage of residues in β-sheet conformation for sealant 1-*cis* in dimer form (red), sealant 1-*trans* in trimer form (blue), sealant 2-*trans* in trimer type1 form (black), sealant 2-*trans* in trimer type2 form (green), sealant 3-*cis* in trimer type1 form (cyan) and sealant 3-*cis* in trimer type2 form (magenta) on simulation data.
4. List S1. Patch residue generated for isopeptide bond formation between Lys and Gln in CHARMM36 force-field.
5. Table S2: Schematic representation of simulations with single cross-linking sites. The bold residues (Lys and Gln pairs) are linked via isopeptide bonds and the next tentative cross-linking residues are marked in *italic* and underlined letters. (H indicates N-terminal ends of the sequences.)
6. Figure S15. Schematic diagram shows the dimer and trimer formation in sealant 1.
7. Figure S16. Schematic diagram shows the two kinds of trimer formation in sealant 2.
8. Figure S17. Schematic diagram shows the two kinds of trimer formation in sealant 3.
9. Figure S18. Determination of *ex-vivo* clotting time of sealants with blood corpuscles by

Hayem method.

1. Figure S19. Determination of clotting time of sealants with plasma by Thrombin clotting

time method experiment.

23. Figure S20. MALDI mass spectrum of model peptide H-Ala-Lys-Ala-Val-OH.

24. Figure S21**.** MALDI mass spectrum of model peptide H-Ala-Gln-His-Val-OH.

25. Figure S22. Extracted ion chromatogram of peptides (EICs) and cross-linked peptide.

26. Figure S23. LC-ESI-MS spectra of model cross-linked peptide.

1. Figure S24. LC-ESI-MS/MS spectra of model cross-linked peptide [precursor ion m/z 824.34 (1+) and 412.50 (2+)].
2. Figure S25. Assignment for the daughter ions arising from the ESI-MS/MS experiment with the model cross-linked peptide.
3. Figure S26**.** ESI-MS spectra of equimolar mixture of two model peptides in absence of TG

and calcium ion.


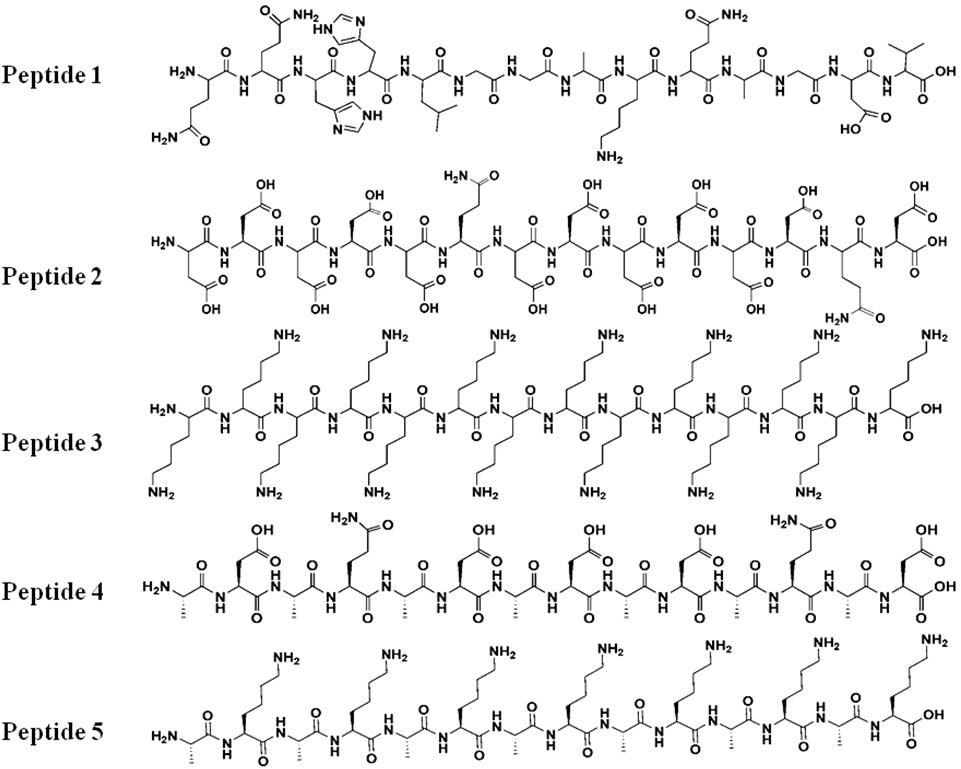


**Figure S1.** Schematic diagram of peptides 1-5.


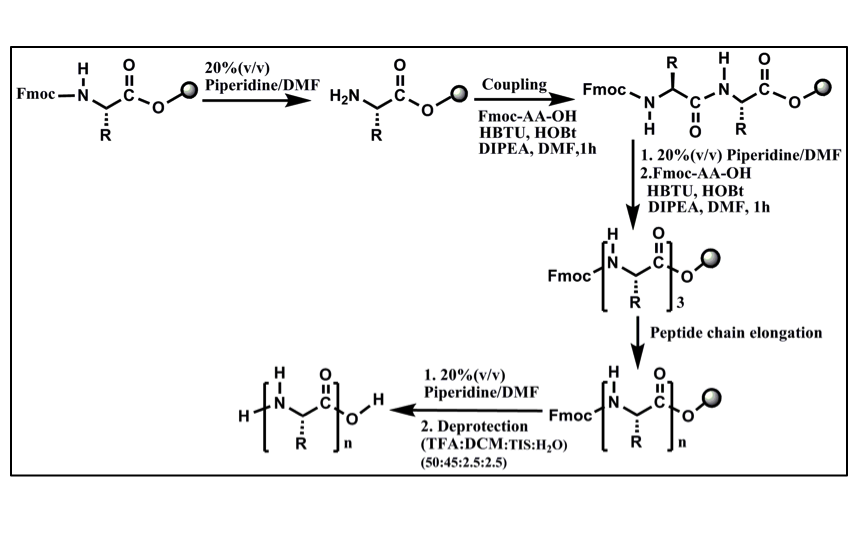


**Figure S2.** General scheme of peptide synthesis.

**Table S1.** Peptide sequences and their mass values

| Peptide code no. | Sequences | Calculated  Mol.Wt. (Da) | Observed Mol.Wt. (Da) | Ion Type |
| --- | --- | --- | --- | --- |
| Peptide 1 | H-Gln-Gln-His-His-Leu-Gly-Gly-Ala-Lys-Gln-Ala-Gly-Asp-Val-OH | 1445.54 | 1446.63 | ([M+H]^+^) |
| Peptide 2 | H-Asp-Asp-Asp-Asp-Asp-Gln-Asp-Asp-Asp-Asp-Asp-Asp-Gln-Asp-OH | 1654.45 | 1655.54,  1678.36,  1693.36 | ([M+H]^+^),  ([M+Na]^+^),  ([M+K]^+^) |
| Peptide 3 | H-Lys-Lys-Lys-Lys-Lys-Lys-Lys-Lys-Lys-Lys-Lys-Lys-Lys-Lys-OH | 1812.43 | 1813.34 | ([M+H]^+^) |
| Peptide 4 | H-^D^Ala-Asp-^D^Ala-Gln-^D^Ala-Asp-^D^Ala-Asp-^D^Ala-Asp-^D^Ala-Gln-^D^Ala-Asp-OH | 1346.52 | 1347.59  1369.45  1385.54 | ([M+H]^+^),  ([M+Na]^+^),  ([M+K]^+^) |
| Peptide 5 | H-^D^Ala-Lys-^D^Ala-Lys-^D^Ala-Lys-^D^Ala-Lys-^D^Ala-Lys-^D^Ala-Lys-^D^Ala-Lys-OH | 1411.94 | 1413.03,  1435.20,  1452.95 | ([M+H]^+^),  ([M+Na]^+^),  ([M+K]^+^) |


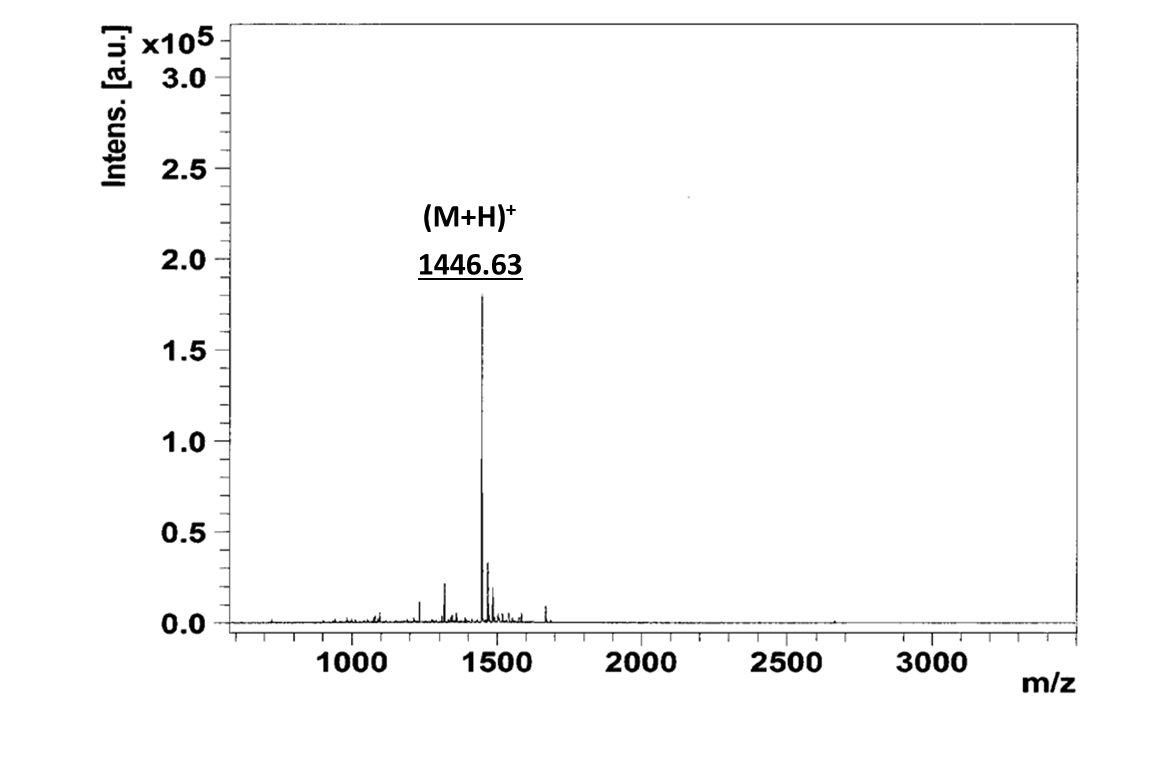


**Figure S3.** MALDI mass spectrum of peptide 1.

Calculated mass: 1445.54 Da ; Observed mass : 1446.63 Da ([M+H]^+^)


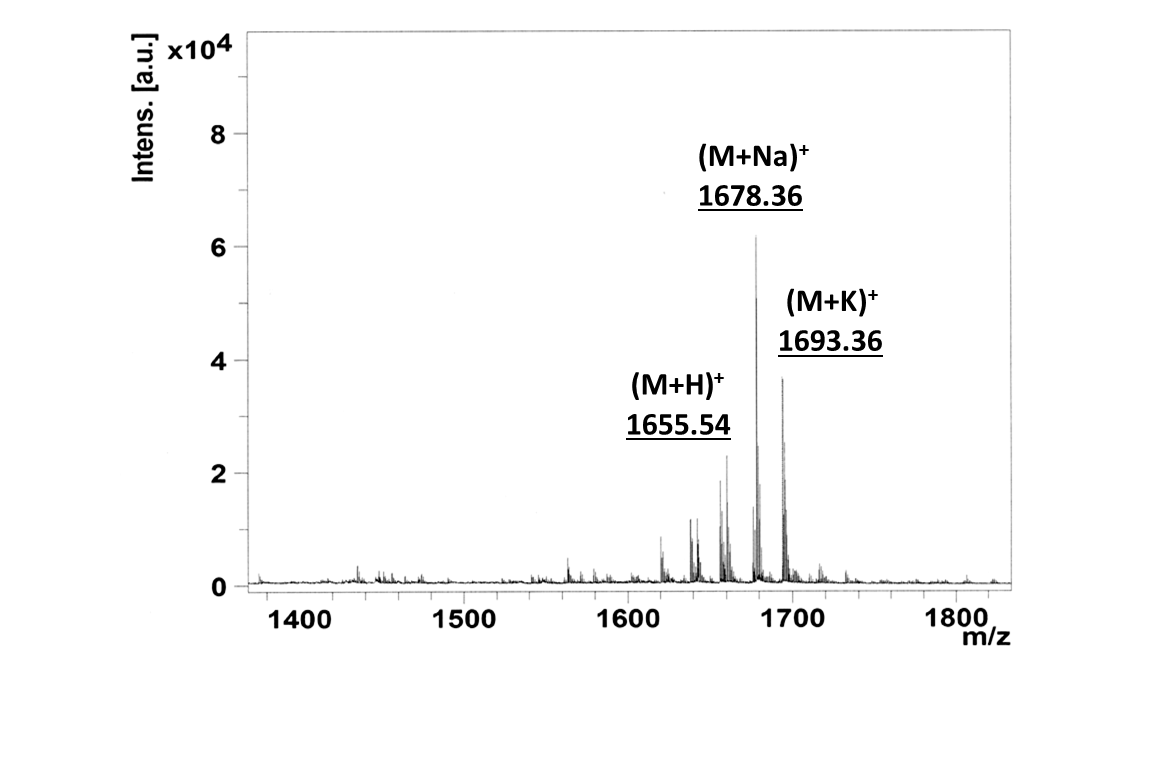


**Figure S4.** MALDI mass spectrum of peptide 2.

Calculated mass: 1654.45 Da; Observed mass : 1655.54 Da ([M+H]^+^), 1678.36 ([M+Na]^+^) and 1693.36 ([M+K]^+^).

**
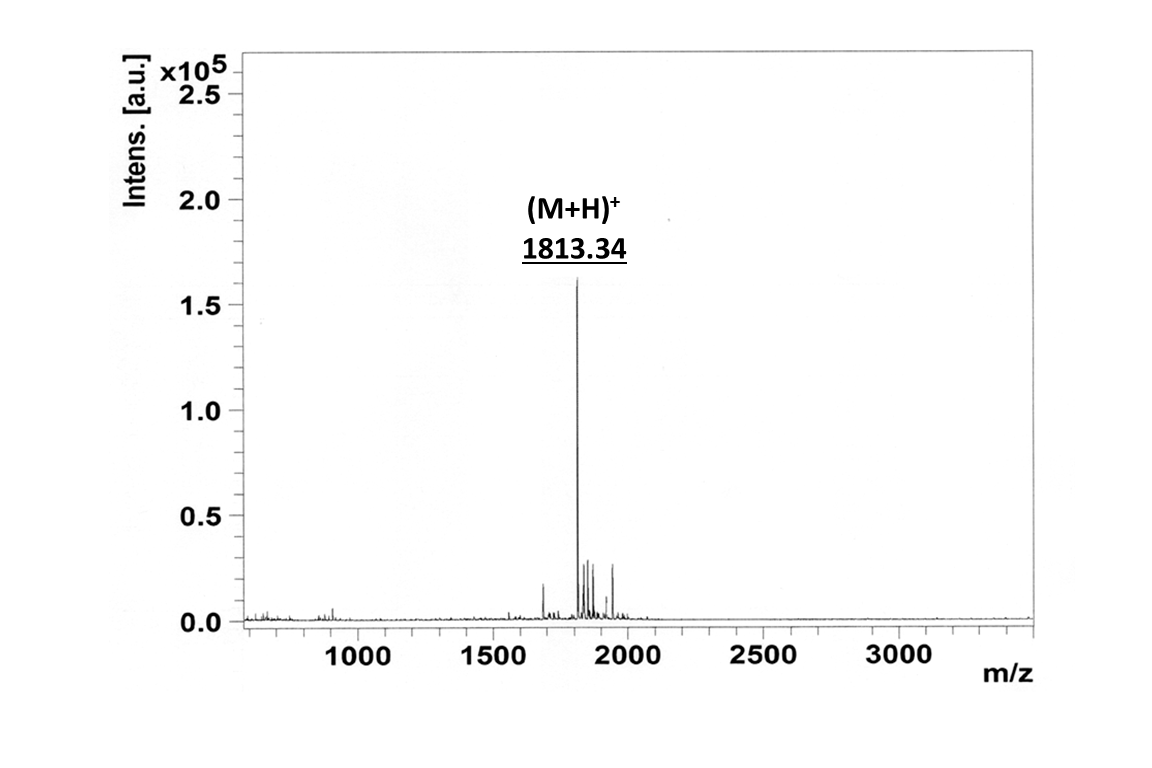
**

**Figure S5.** MALDI mass spectrum of peptide 3.

Calculated mass: 1812.43 Da ; Observed mass : 1813.34 Da ([M+H]^+^).

**
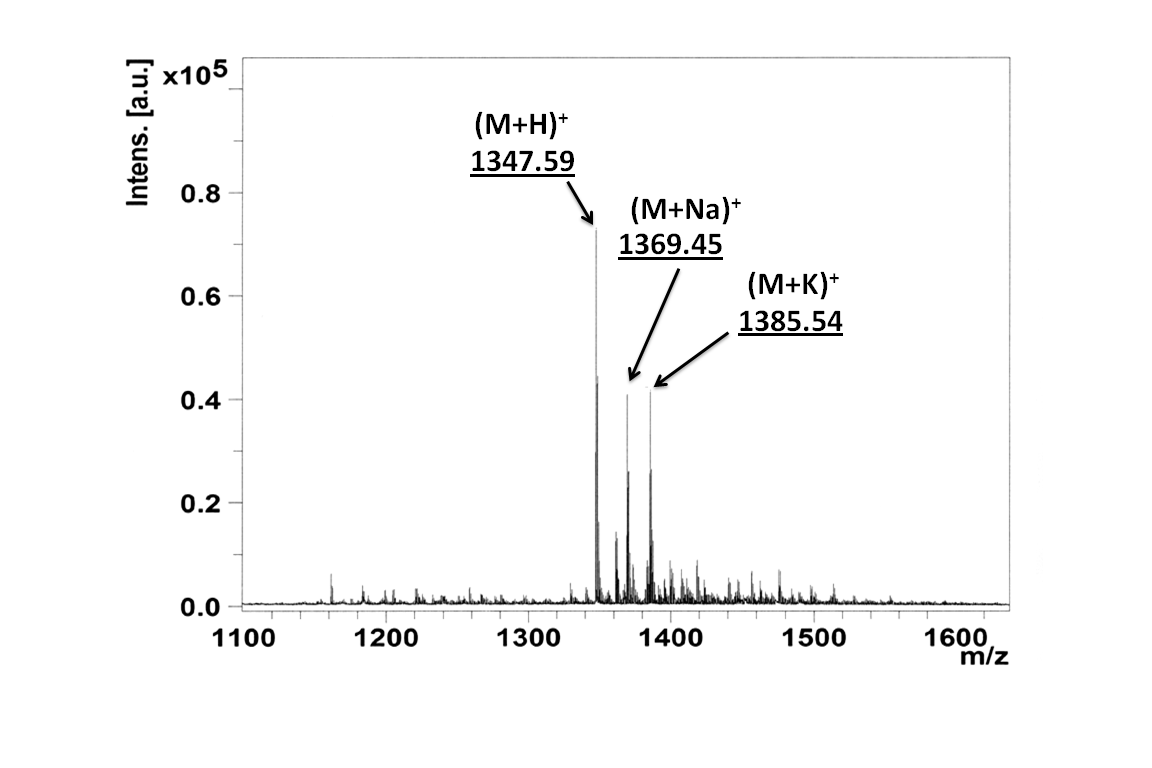
**

**Figure S6.** MALDI mass spectrum of peptide 4.

Calculated mass: 1346.52 Da; Observed mass : 1347.59 Da ([M+H]^+^), 1369.45 ([M+Na]^+^) and 1385.54 ([M+K]^+^).

**
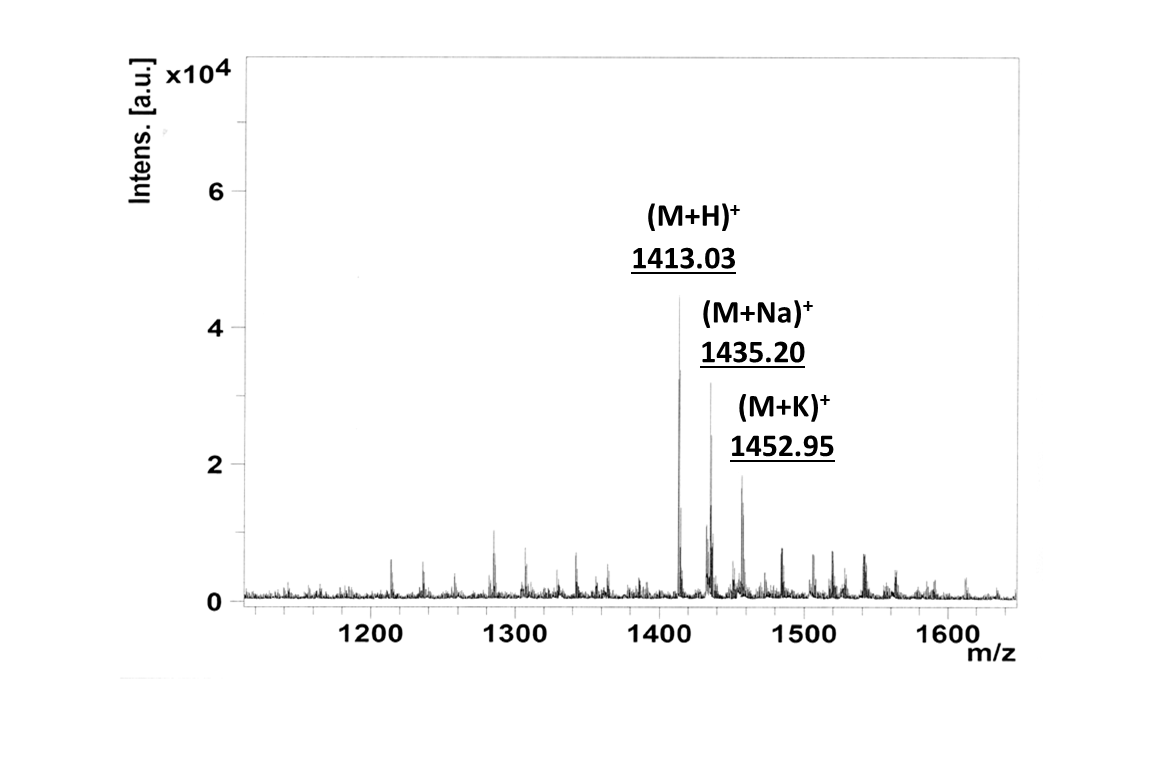
**

**Figure S7.** MALDI mass spectrum of peptide 5.

Calculated mass: 1411.94 Da ; Observed mass : 1413.03 Da ([M+H]^+^), 1435.20 ([M+Na]^+^) and 1452.95 ([M+K]^+^).


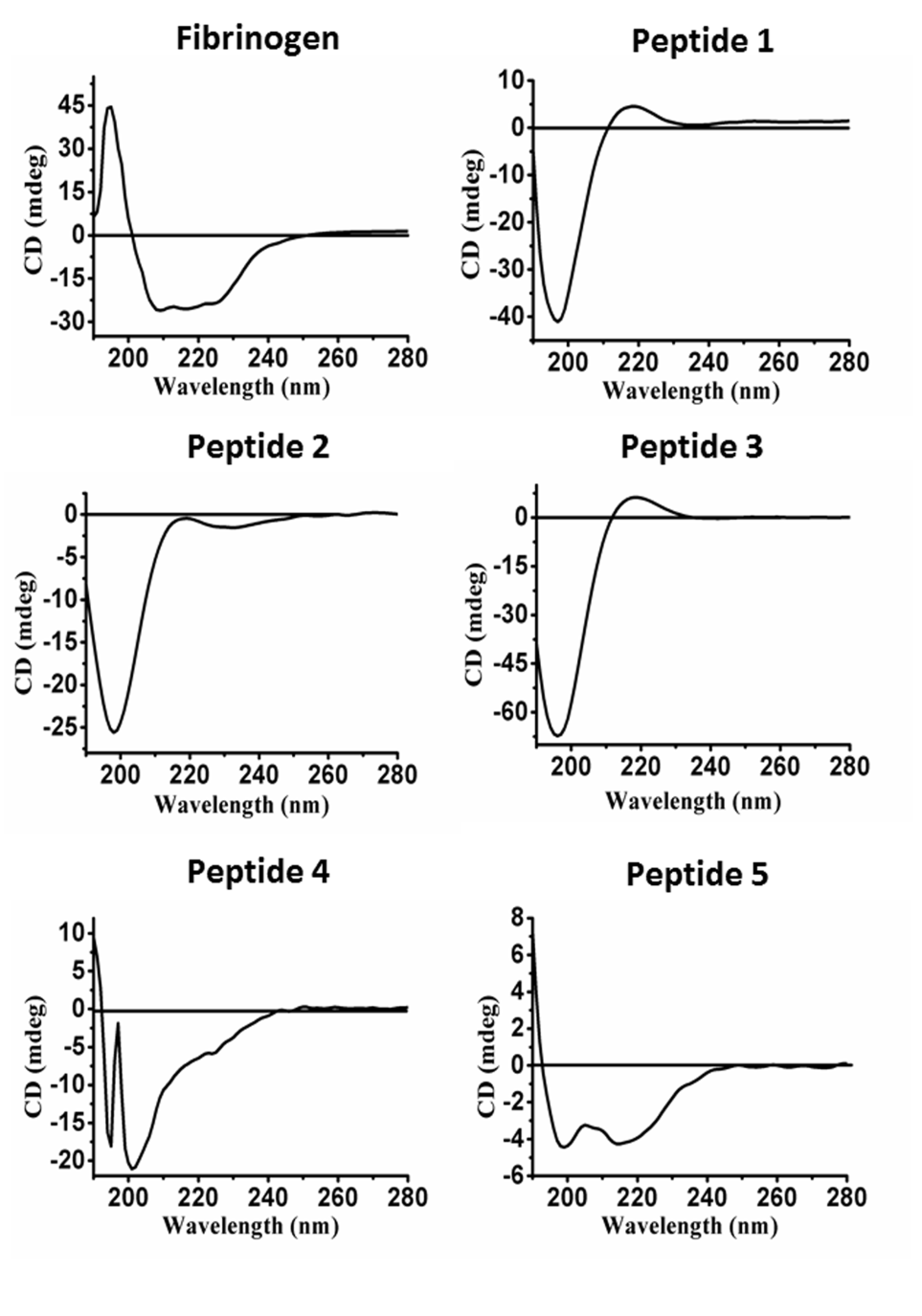


**Figure S8.** CD spectra of fibrinogen and peptides 1-5 in 50 mM Tris HCl, pH 7.4 buffer.


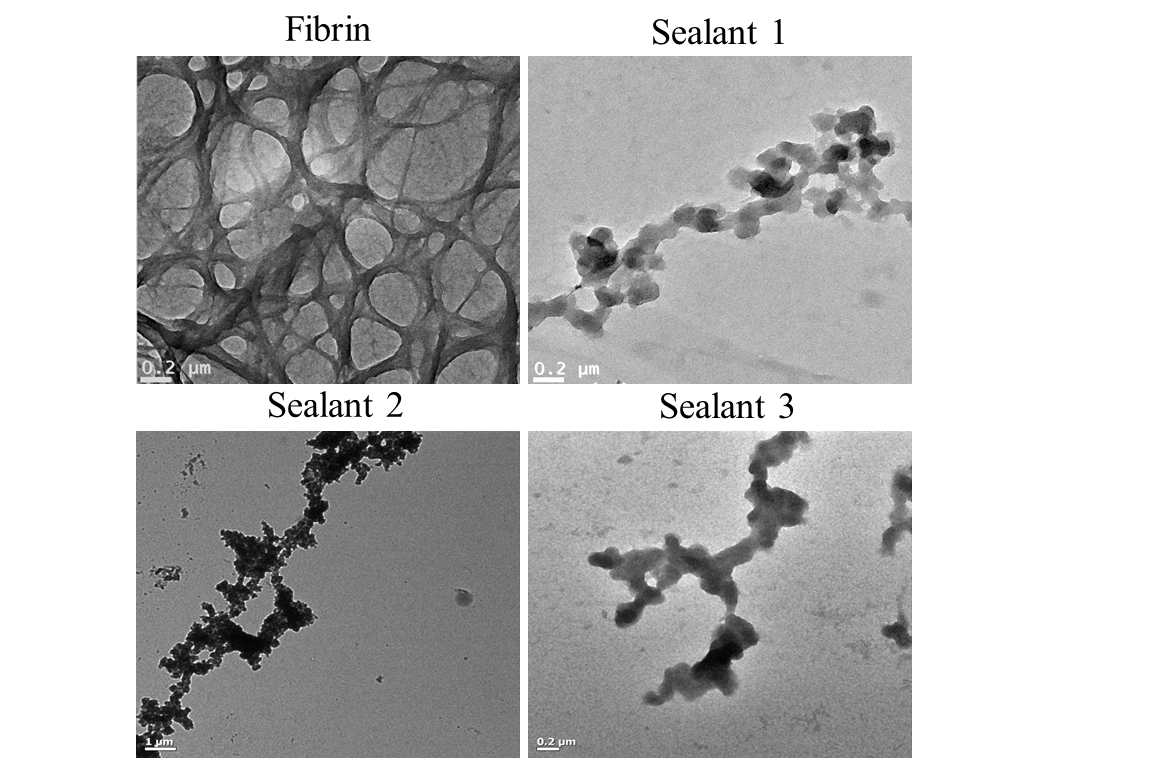


**Figure S9.** TEM images of Fibrin, sealants 1, 2 and 3.


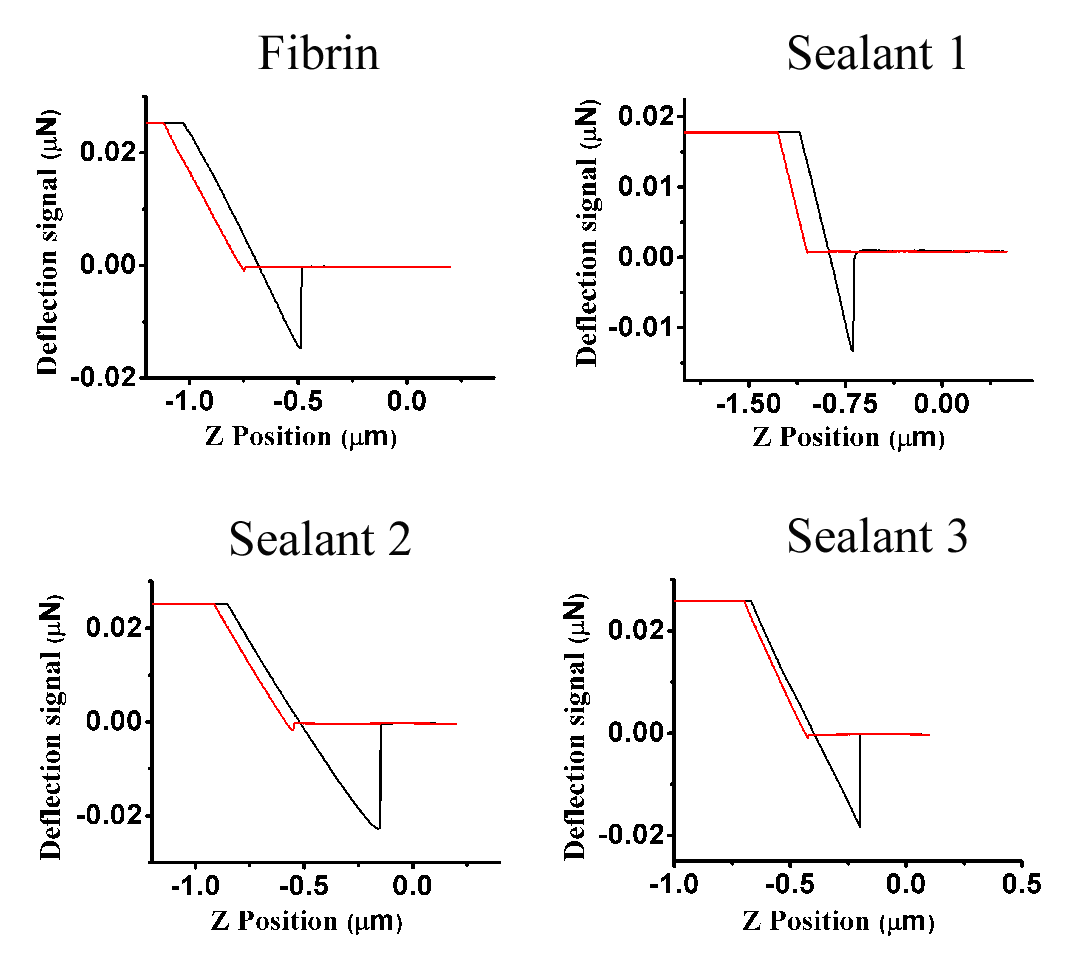


**Figure S10.** Force–distance curves for fibrin, sealants 1, 2 and 3 showing attraction and repulsion using silicon nitride tip.

(a)


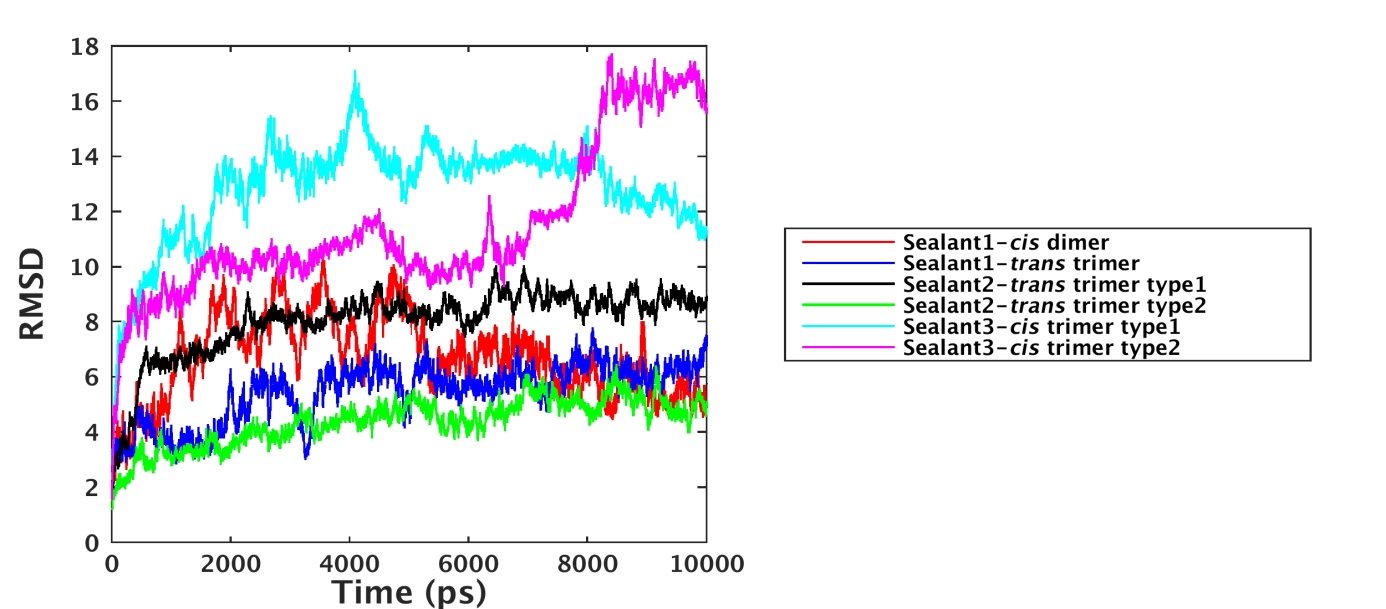


(b)


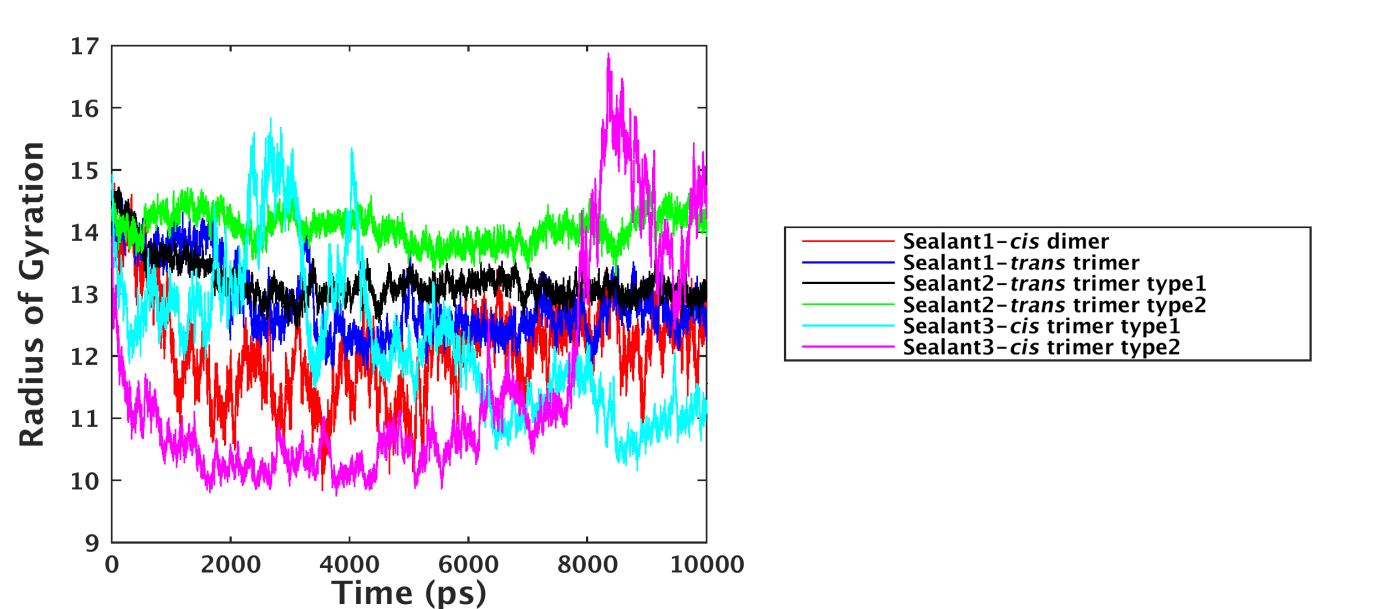


**Figure S11.** Measurement of structural fluctuation : (a) Root-Mean-Square Deviation (Å) and (b) Radius of Gyration (Å) of the sealant 1-*cis* in dimer form (red), sealant 1-*trans* in trimer form (blue), sealant 2-*trans* in trimer type1 form (black), sealant 2-*trans* in trimer type2 form (green), sealant 3-*cis* in trimer type1 form (cyan) and sealant 3-*cis* in trimer type2 form (magenta) on simulation data.


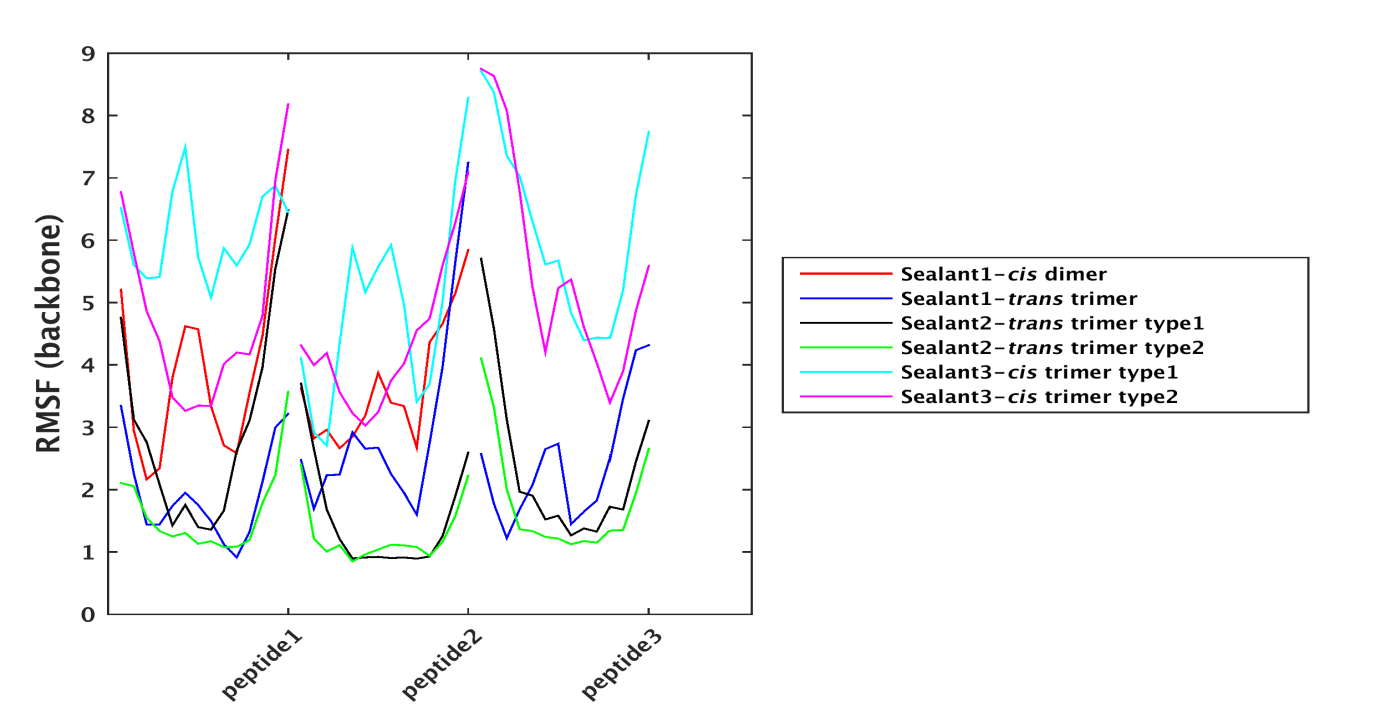


**Figure S12.** Root-Mean-Square Fluctuation (RMSF) (Å) of individual residues in all peptides taking into consideration only backbone atoms of the residues for sealant 1-*cis* in dimer form (red), sealant 1-*trans* in trimer form (blue), sealant 2-*trans* in trimer type1 form (black), sealant 2-*trans* in trimer type2 form (green), sealant 3-*cis* in trimer type1 form (cyan) and sealant 3-*cis* in trimer type2 form (magenta) on simulation data.


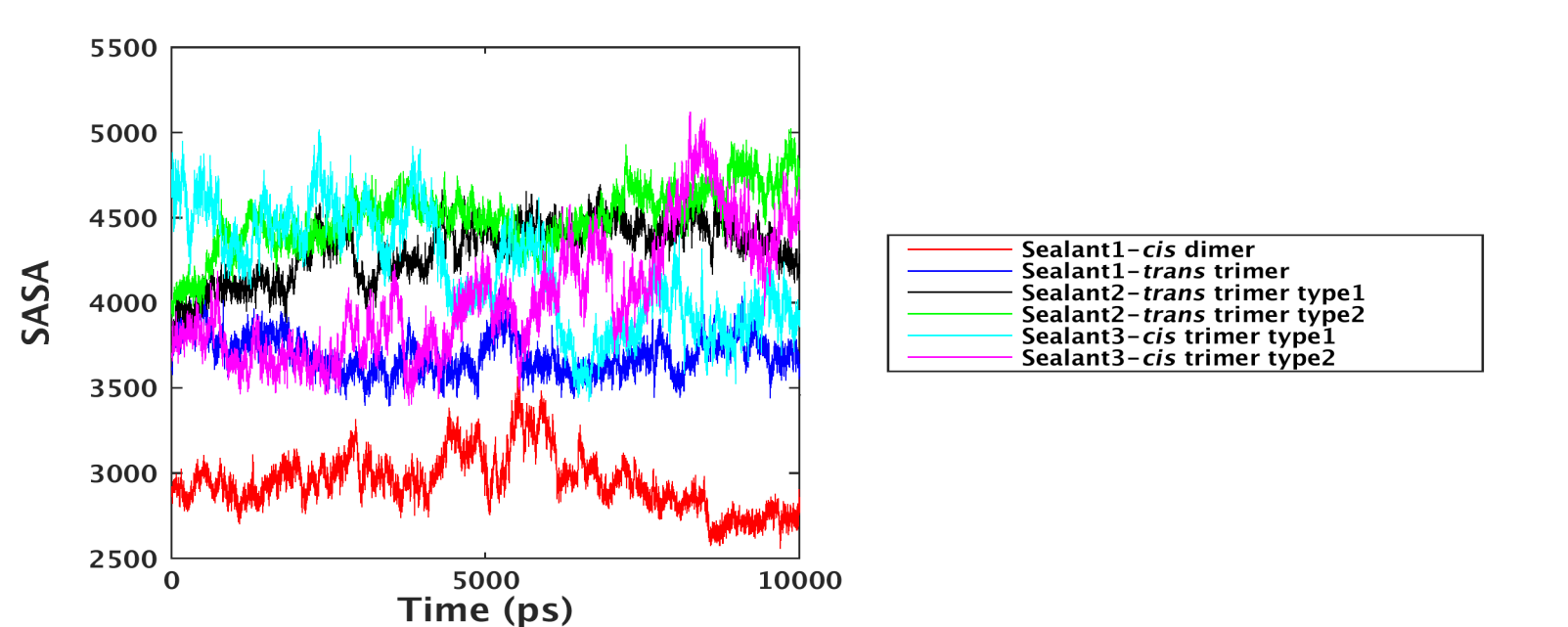


**Figure S13.** Time evolution plot of Solvent Accessible Surface Area (SASA) in Å^2^ for each sealant. For sealant pair 1 in dimer form (red), sealant pair 1 in trimer form (blue), sealant 2-*trans* in trimer type1 form (black), sealant 2-*trans* in trimer type2 form (green), sealant 3-*cis* in trimer type1 form (cyan) and sealant 3-*cis* in trimer type2 form (magenta) on simulation data.


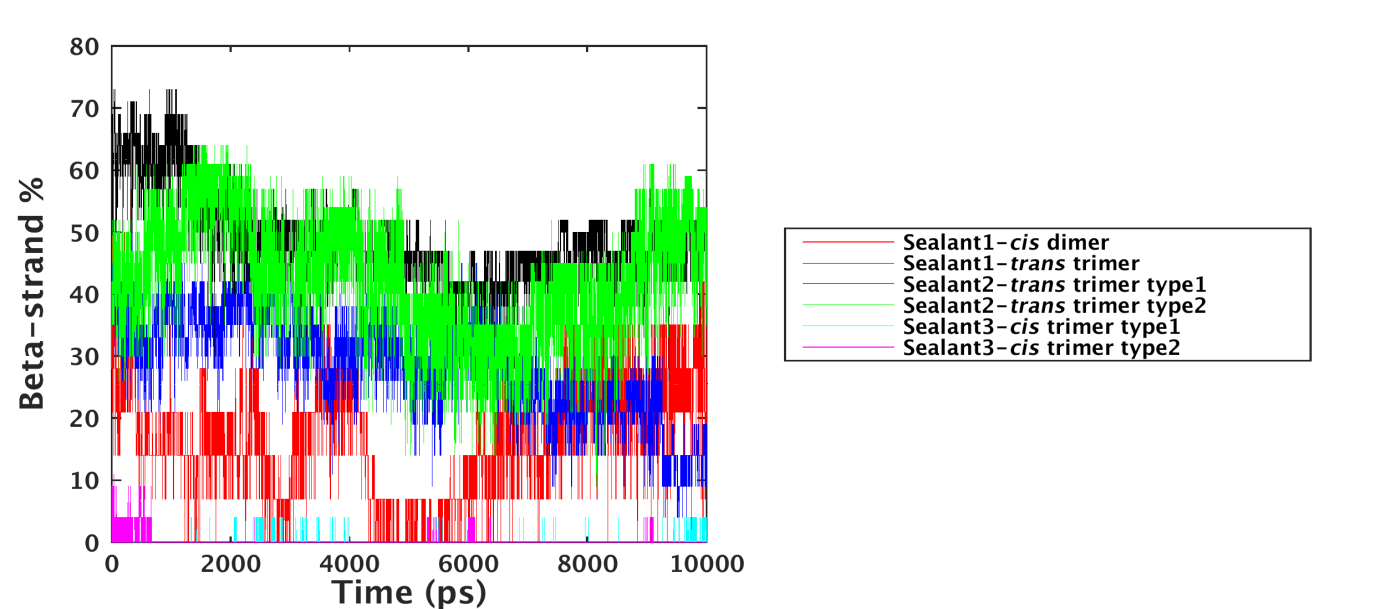


**Figure S14.**  Time evolution plot of secondary structural element: percentage of residues in β-sheet conformation for sealant 1-*cis* in dimer form (red), sealant 1-*trans* in trimer form (blue), sealant 2-*trans* in trimer type1 form (black), sealant 2-*trans* in trimer type2 form (green), sealant 3-*cis* in trimer type1 form (cyan) and sealant 3-*cis* in trimer type2 form (magenta) on simulation data.

**List S1.** Patch residue generated for isopeptide bond formation between Lys and Gln in CHARMM36 force-field.

PRES IPEP 0.00 ! patch for isopeptide bond

GROUP

ATOM 1CD CT2 -0.18

ATOM 1HD1 HA2 0.11

ATOM 1HD2 HA2 0.11

ATOM 1CE CT2 -0.18

ATOM 1HE1 HB2 0.11

ATOM 1HE2 HB2 0.11

GROUP

ATOM 2CD C 0.51

ATOM 2OE1 O -0.51

ATOM 2NE2 NH1 -0.47

ATOM 2HE22 H 0.31

ATOM 2CG CT2 -0.14

ATOM 2HG1 HA2 0.11

ATOM 2HG2 HA2 0.11

DELETE ATOM 1HZ1

DELETE ATOM 1HZ2

DELETE ATOM 1HZ3

DELETE ATOM 1NZ

DELETE ATOM 2HE21

BOND 1CE 2NE2

angle 1ce 2ne2 2cd

angle 1ce 2ne2 2he22

dihe 1ce 2ne2 2cd 2cg

dihe 1cd 1ce 2ne2 2cd

impr 2ne2 2cd 1ce 2he22

| Sealant code no. | Sequence |
| --- | --- |
|  |  |
| Sealant 1-*cis*  (orientation1) | H-**Gln**-Gln-His-His-Leu-Gly-Gly-Ala-*Lys*-Gln-Ala-Gly-Asp-Val-OH  HO-Val-Asp-Gly-Ala-Gln-**Lys**-Ala-Gly-Gly-Leu-His-His-Gln-*Gln*-H |
| Sealant 1-*trans*  (orientation2) | H-Gln-**Gln**-His-His-Leu-Gly-Gly-Ala-*Lys*-Gln-Ala-Gly-Asp-Val-OH  HO-Val-Asp-Gly-Ala-Gln-**Lys**-Ala-Gly-Gly-Leu-His-His-*Gln*-Gln-H |
|  |  |
| Sealant 2-*trans* | H-Asp-Asp-Asp-Asp-Asp-**Gln**-Asp-Asp-Asp-Asp-Asp-Asp-*Gln*-Asp-OH  HO-Lys-**Lys**-Lys-Lys-Lys-Lys-Lys-Lys-Lys-Lys-Lys-Lys-Lys-Lys-H |
|  |  |
| Sealant 3-*cis* | H-^D^Ala-Asp-^D^Ala-**Gln**-^D^Ala-Asp-^D^Ala-Asp-^D^Ala-Asp-^D^Ala-*Gln*-^D^Ala-Asp-OH  HO- Lys-^D^Ala- **Lys**-^D^Ala- Lys-^D^Ala- Lys-^D^Ala- Lys-^D^Ala- Lys-^D^Ala- Lys-^D^Ala-H |

**Table S2.** Schematic representation of simulations with single cross-linking sites. The bold residues (Lys and Gln pairs) are linked via isopeptide bonds and the next tentative cross-linking residues are marked in *italic* and underlined letters. (H indicates N-terminal ends of the sequences.)


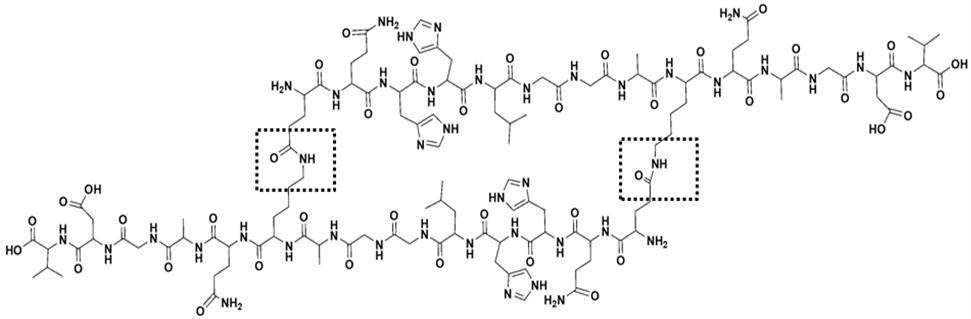


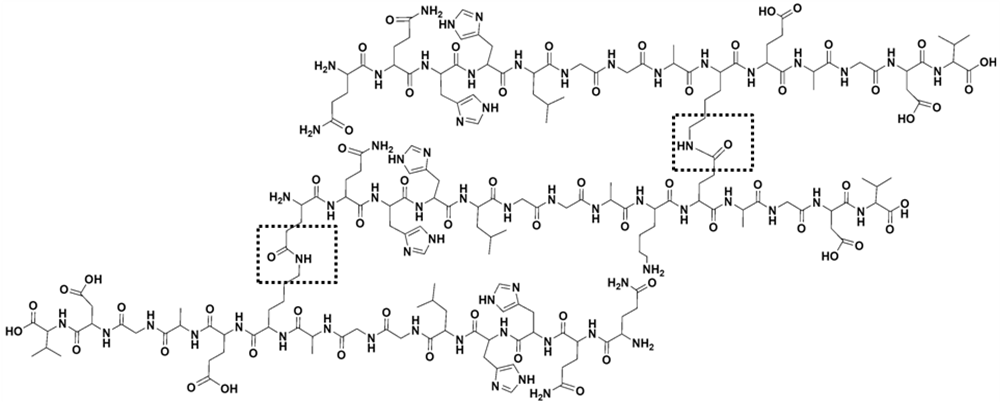


**Figure S15.** Schematic diagram shows isopeptide bond (marked with dashed box) mediated dimer and trimer formation in sealant 1.


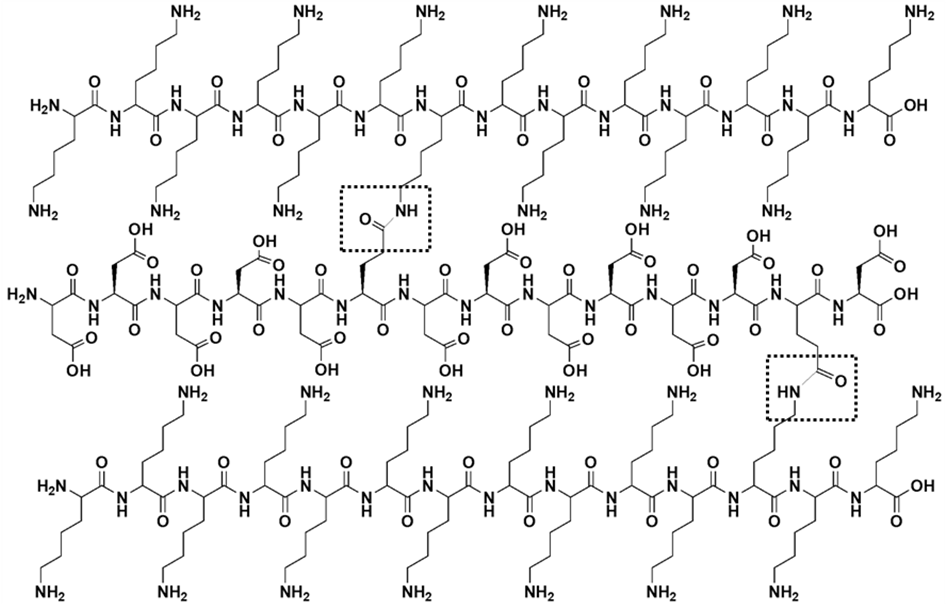


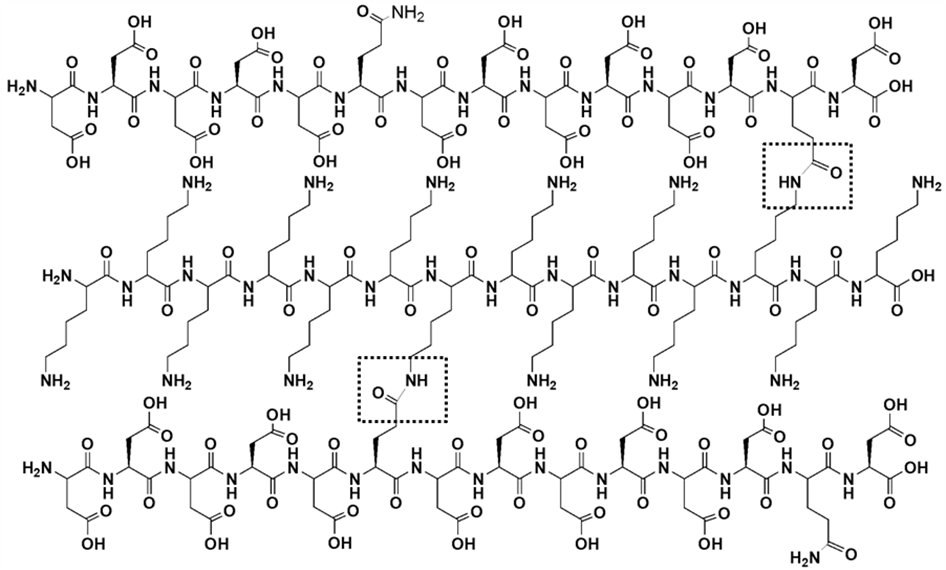


**Figure S16.** Schematic diagram shows the isopeptide bond (marked in the dashed box) mediated two kinds of trimer (2 units of peptide 2 and 1 unit of peptide 3 and vice versa) formation in sealant 2.


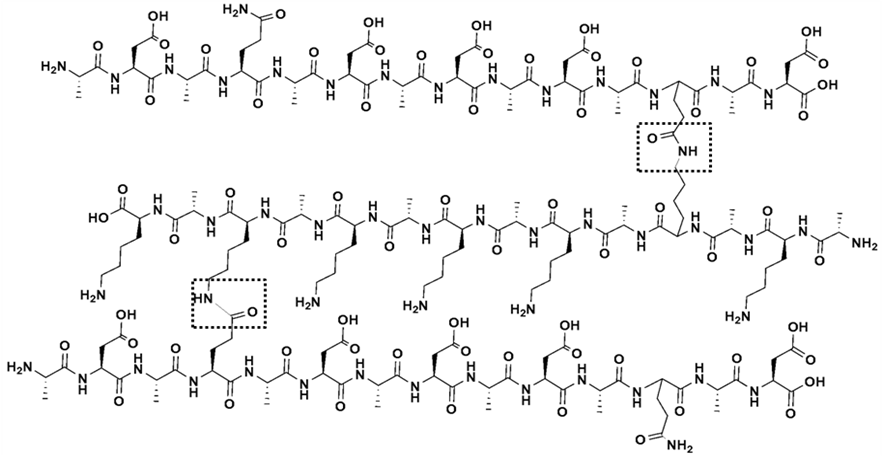


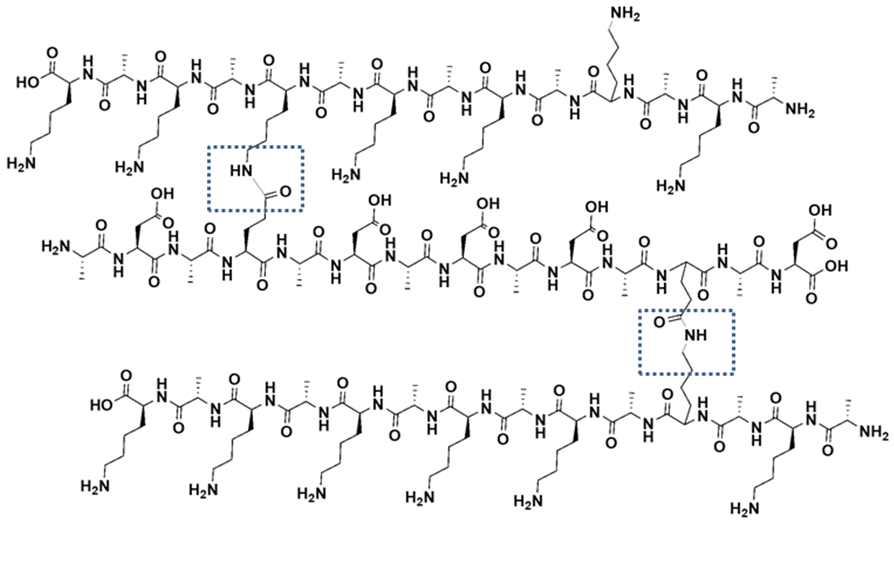


**Figure S17.** Schematic diagram shows the isopeptide bond (marked with dashed box) mediated two kinds of trimer (2 units of peptide 4 and 1 unit of peptide 5 and vice versa) formation in sealant 3.


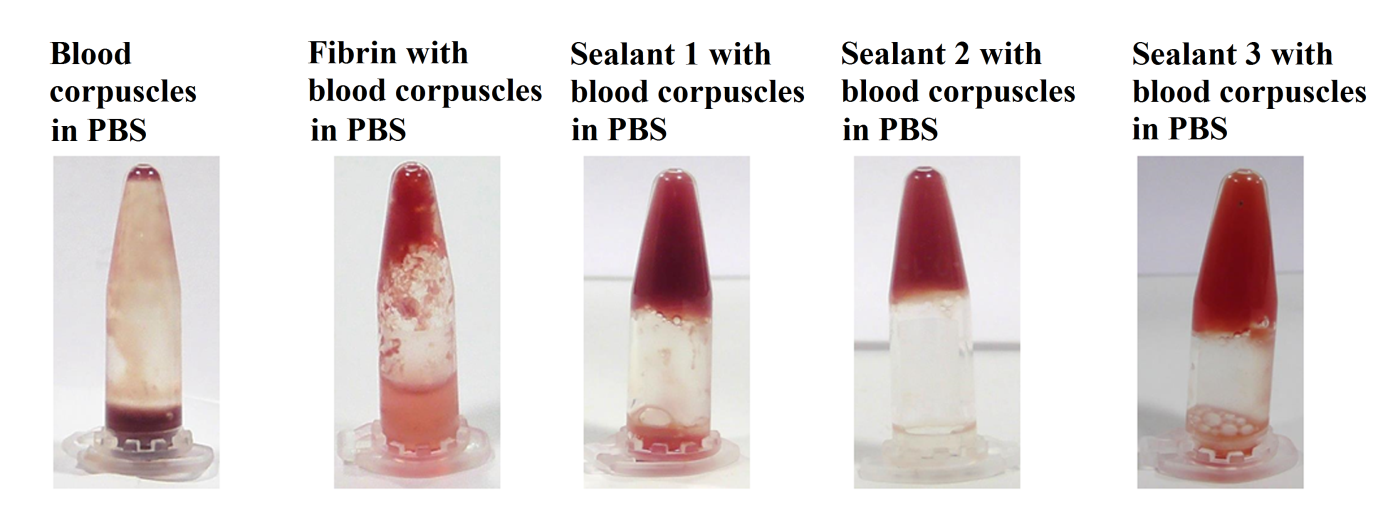


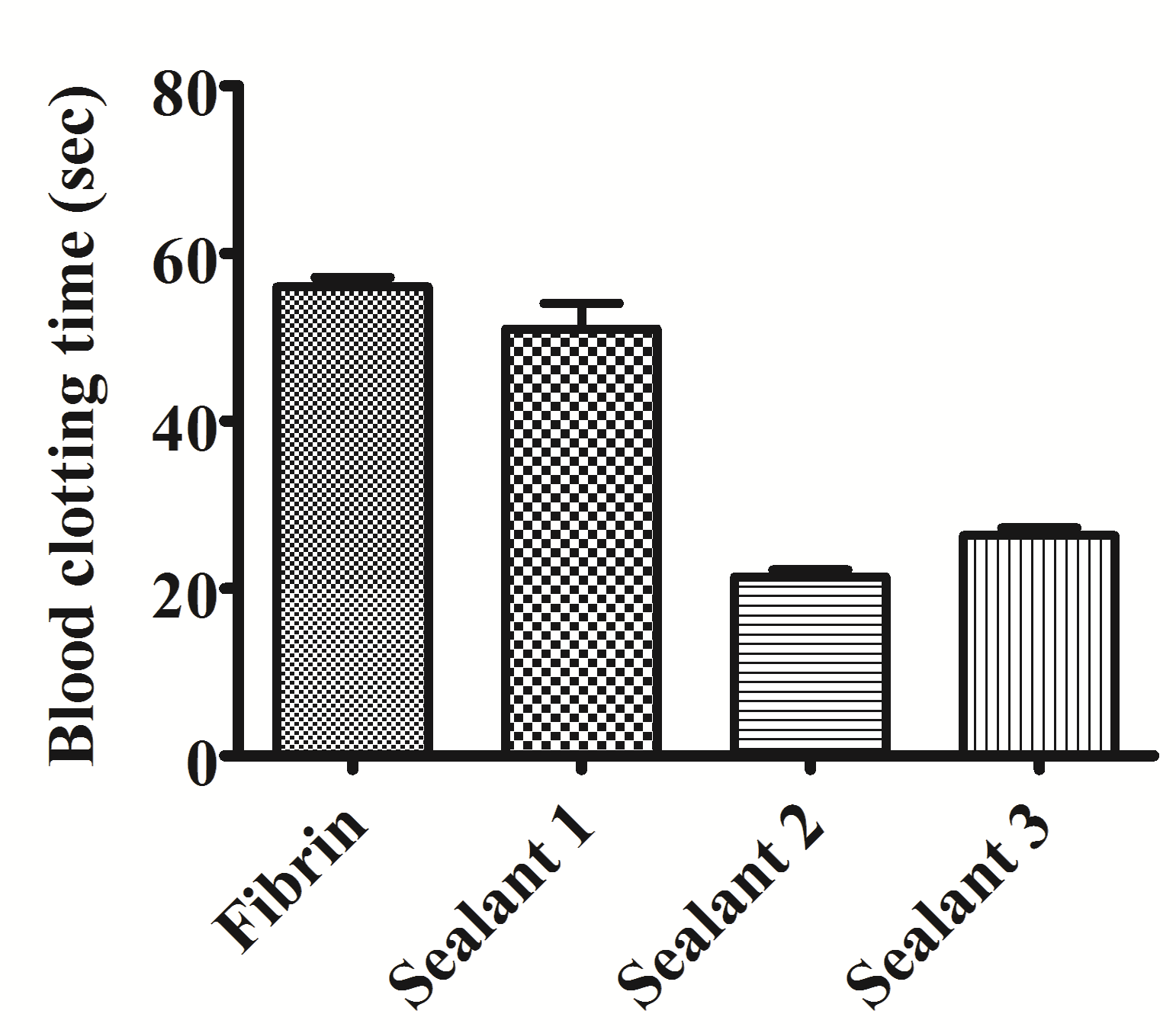


**Figure S18.**  Determination of *ex-vivo* clotting time of sealants with blood corpuscles by Hayem method. Blood corpuscles were taken in PBS and sealants were added to this solution externally in order to measure clotting time. Fibrin, sealants 1, 2 and 3 clot blood corpuscles in 56±2, 51±5.3, 21.3±1.5 and 26.3±1.5 secs, respectively.


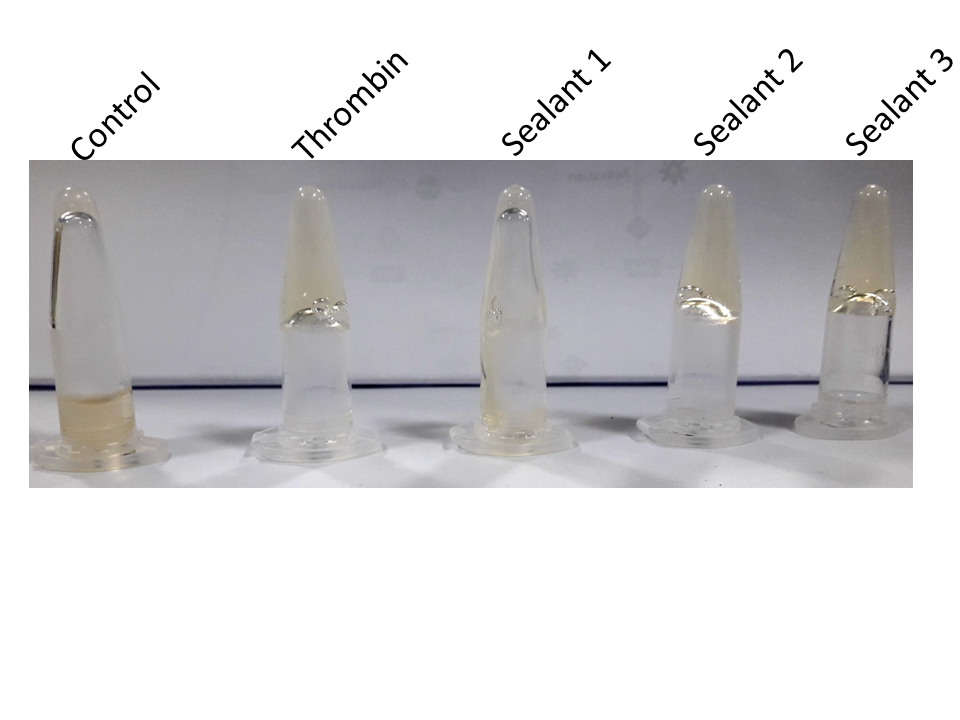


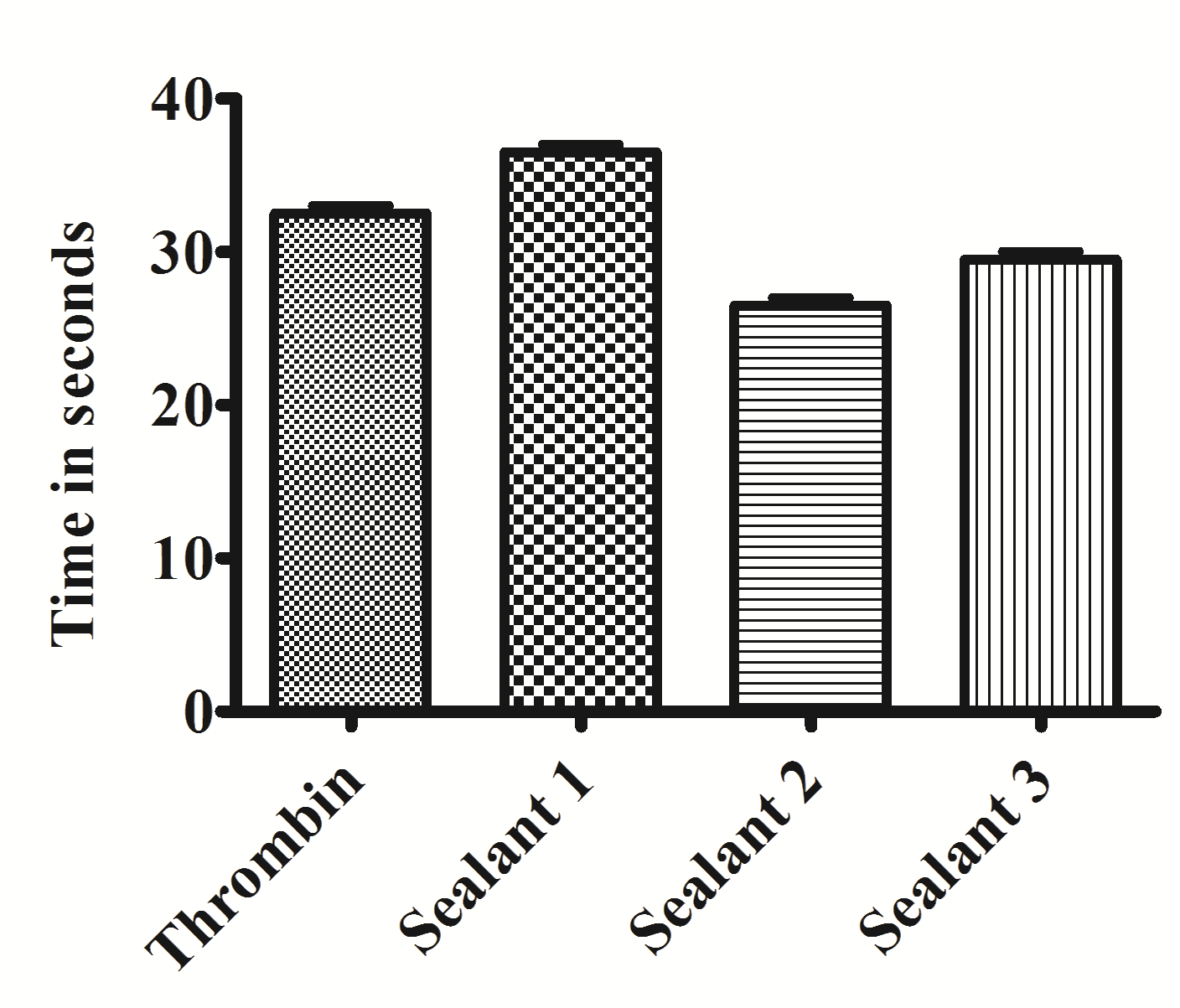


**Figure S19.** Determination of clotting time of sealants with plasma by thrombin clotting time method. The clotting time in presence of thrombin, sealants 1, 2 and 3 are 32±2, 35±1, 25±1 and 28±2 secs, respectively.


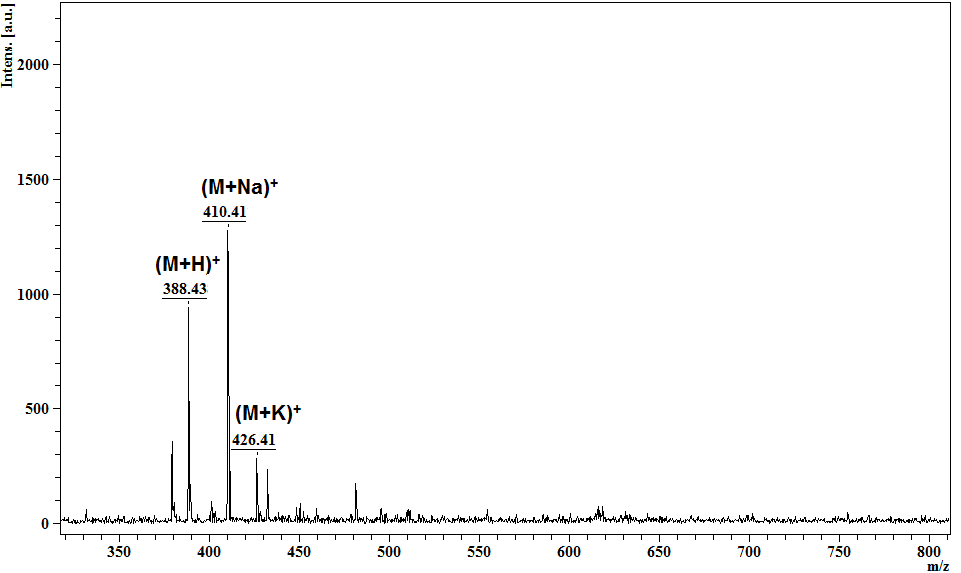


**Figure S20.** MALDI mass spectrum of model peptide H-Ala-Lys-Ala-Val-OH.

Calculated mass: 387.41 Da ; Observed mass : 388.43 Da ([M+H]^+^), 410.41 Da ([M+Na]^+^) and 426.41 Da ([M+K]^+^).


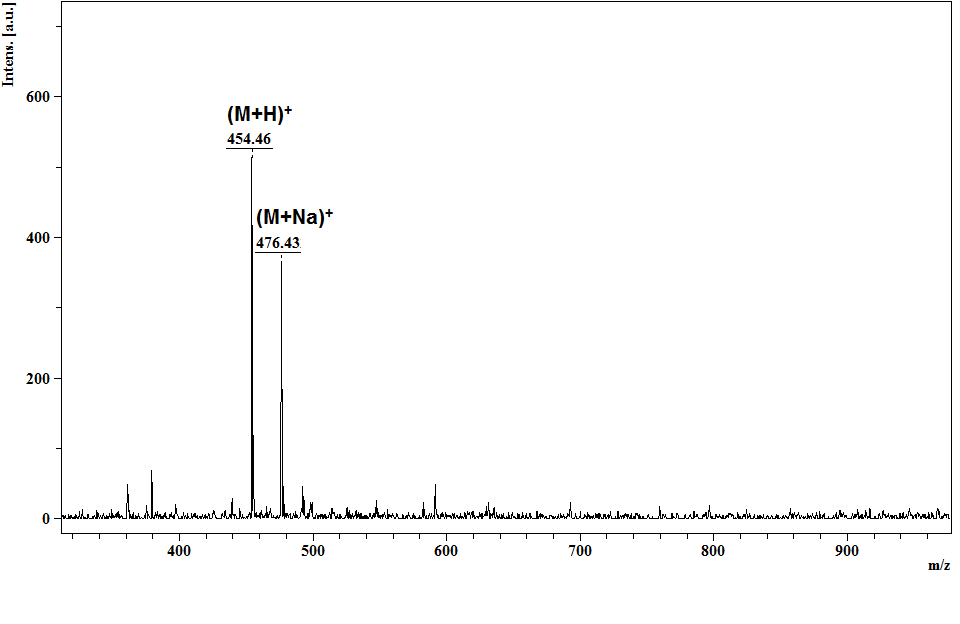


**Figure S21.** MALDI mass spectrum of model peptide H-Ala-Gln-His-Val-OH.

Calculated mass: 453.43 Da ; Observed mass : 454.46 Da ([M+H]^+^), 476.43 Da ([M+Na]^+^).


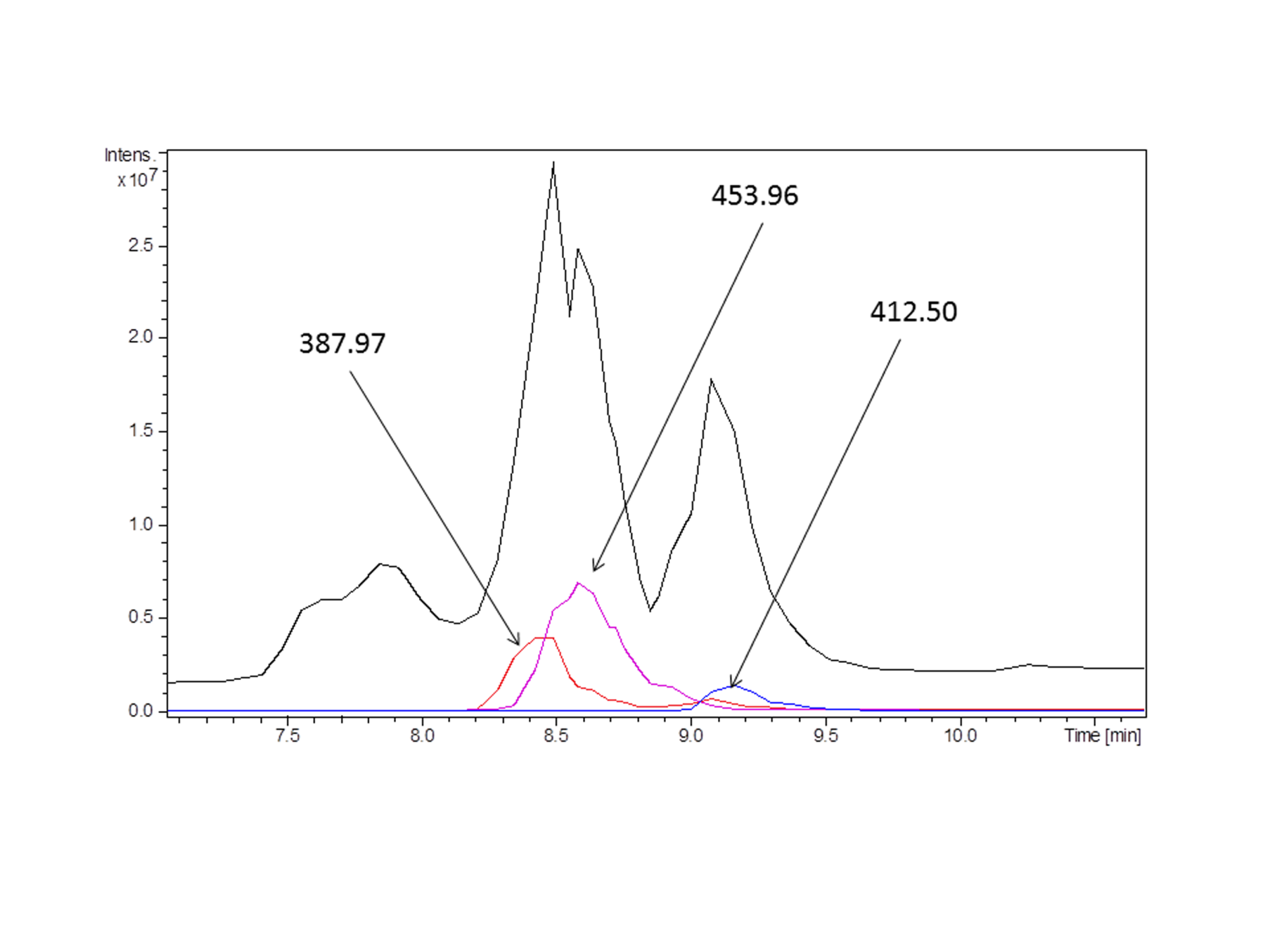


**Figure S22.** Extracted ion chromatograms of peptides (EICs) and cross-linked peptide. (black) Total ion chromatogram from the LC-ESI MS run. The three EICs are obtained by selecting the m/z values indicated against the traces. 412.50 is the 2+ charge state of the cross-linked peptide.


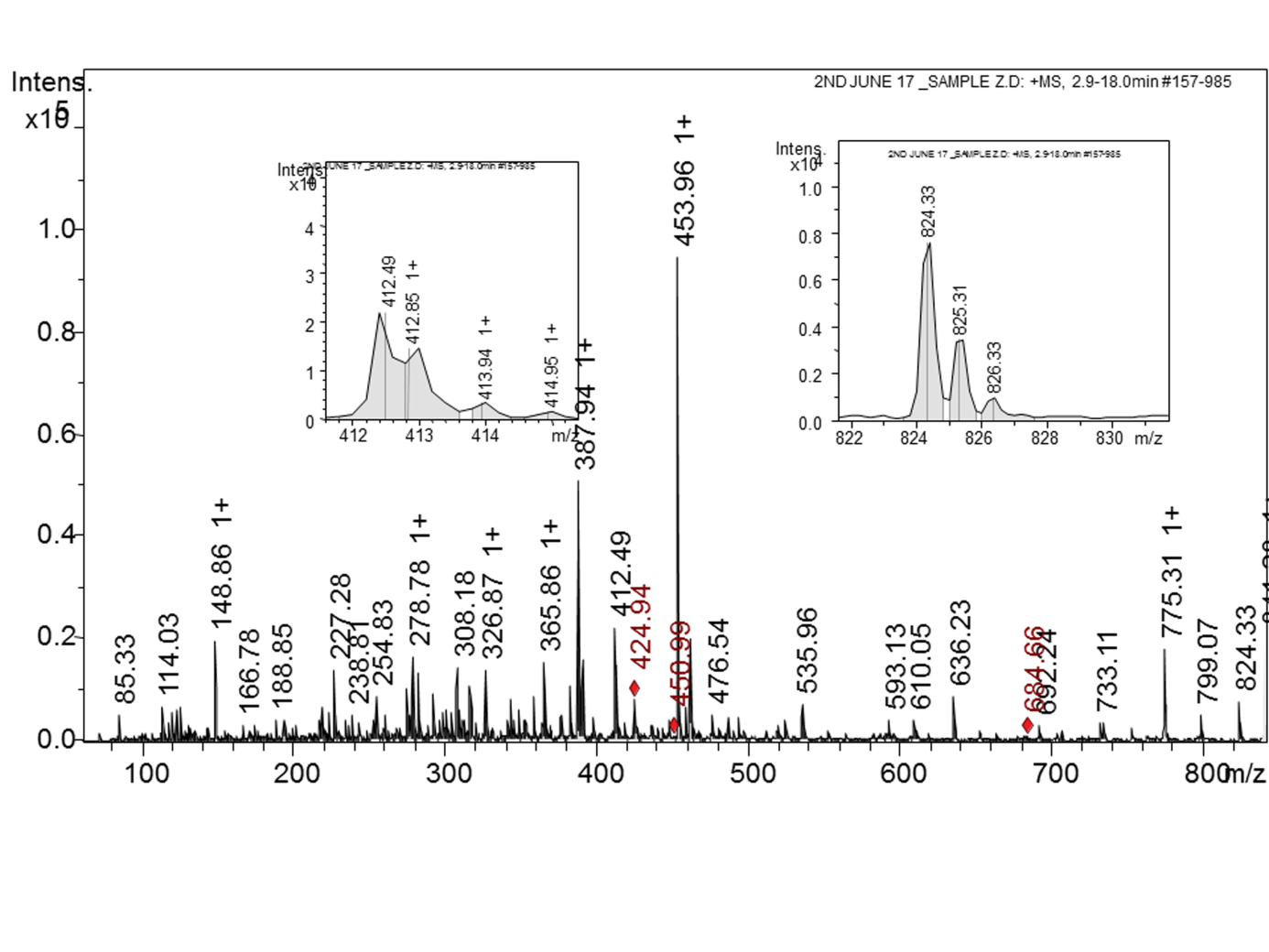


**Figure S23.** LC-ESI-MS spectra of model cross-linked peptide. The isotopic patterns of model cross-linked peptide [precursor ion m/z 412.50 (2+) and 824.34 (1+)] are shown in the inset.


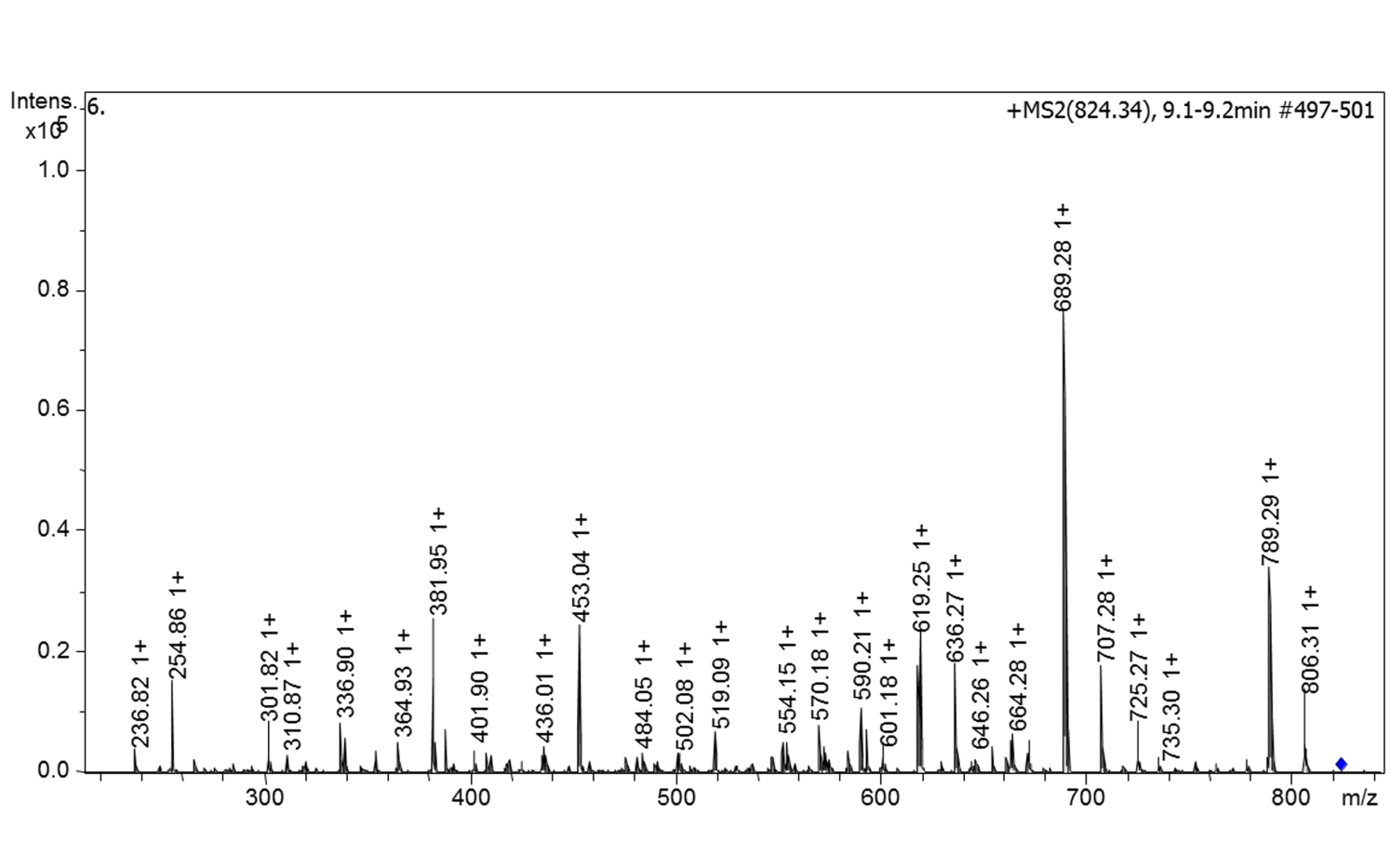


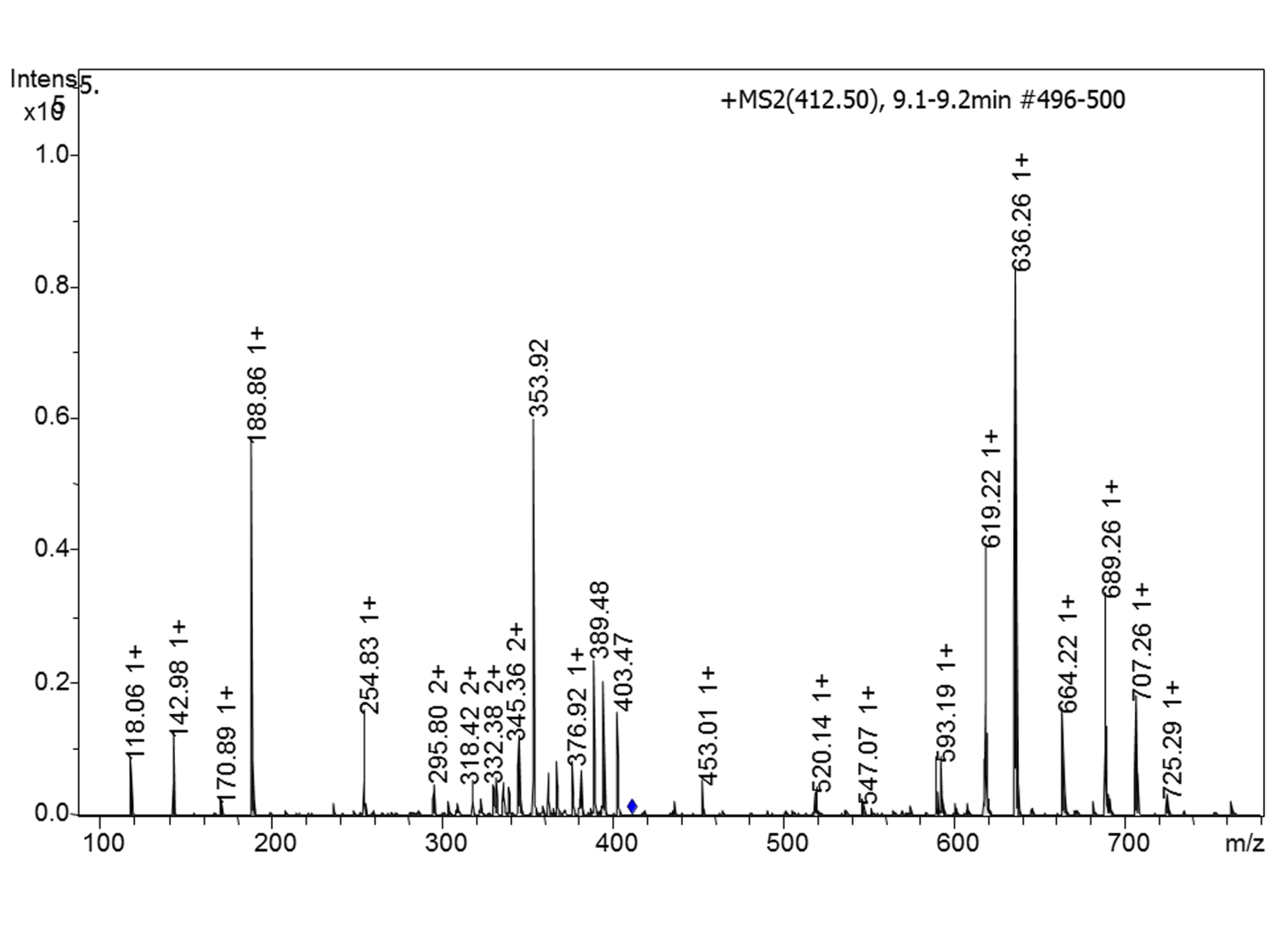
 **Figure S24.** LC-ESI-MS/MS spectra of model cross-linked peptide [precursor ion m/z 824.34 (1+) and 412.50 (2+)].


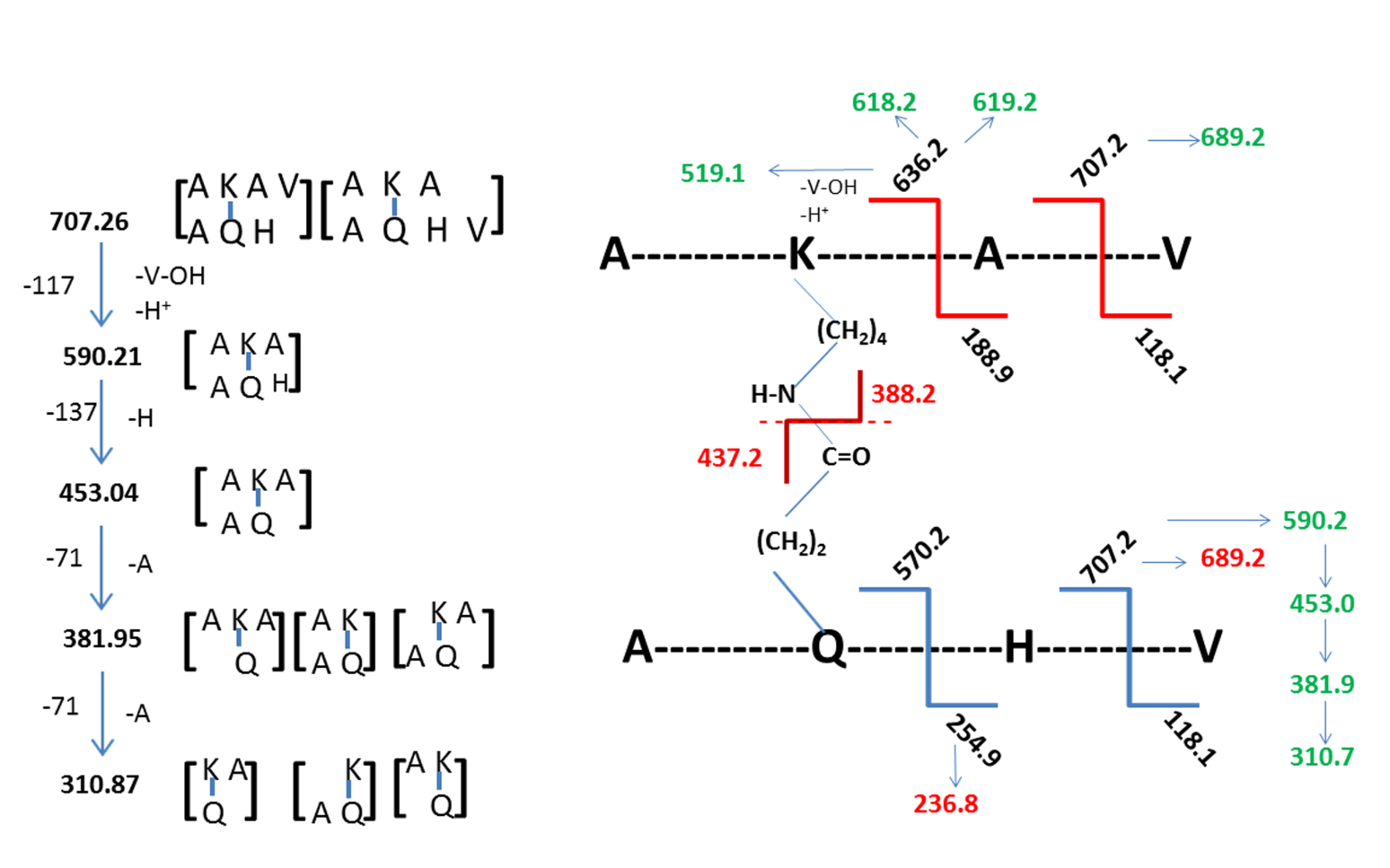


**Figure S25.** Assignment for the daughter ions arising from the ESI-MS/MS experiment with the model cross-linked peptide. In a cross-linked peptide cleavages in both segments can happen and therefore the b/y ion nomenclature is confusing. So we are showing the cleavage pattern in the diagram only.The peaks arising from neutral losses of NH_3_ (619.2 Da) and H_2_O (689.2 Da, 236.8 Da) are shown in green and red for two peptide chains.

**Figure S26.** ESI-MS spectra of equimolar mixture of two model peptides in absence of TG and calcium ion was recorded. Expected molecular weight of model peptide H-Ala-Lys-Ala-Val-OH is 387.41 Da and the molecular weight of model peptide H-Ala-Gln-His-Val-OH is 453.31 Da. ESI- MS spectra supports the role of TG in peptide cross-linking. This experiment was performed as control experiment.
